# Supplementary material for: The complex genetic architecture of shoot growth natural variation in Arabidopsis thaliana
Source: PLoS Genet. 2019 Apr 22;15(4):e1007954. doi: 10.1371/journal.pgen.1007954 (PMC6476473; doi:10.1371/journal.pgen.1007954)
Supplement: S1 Fig — Frequency histograms showing the distributions of the PRA29, RER16-29 and Compactness29 traits within each of the four RIL sets in well-watered (WW: blue) and water deficit (WD: orange) conditions. Phenotypic values for parental accessions from these specific experiments are indicated by blue (WW) and red (WD) ticks (see inset for legend) just above the x axes. (PDF) [file pgen.1007954.s001.pdf]

>>> SUPPLEMENTARY MATERIAL

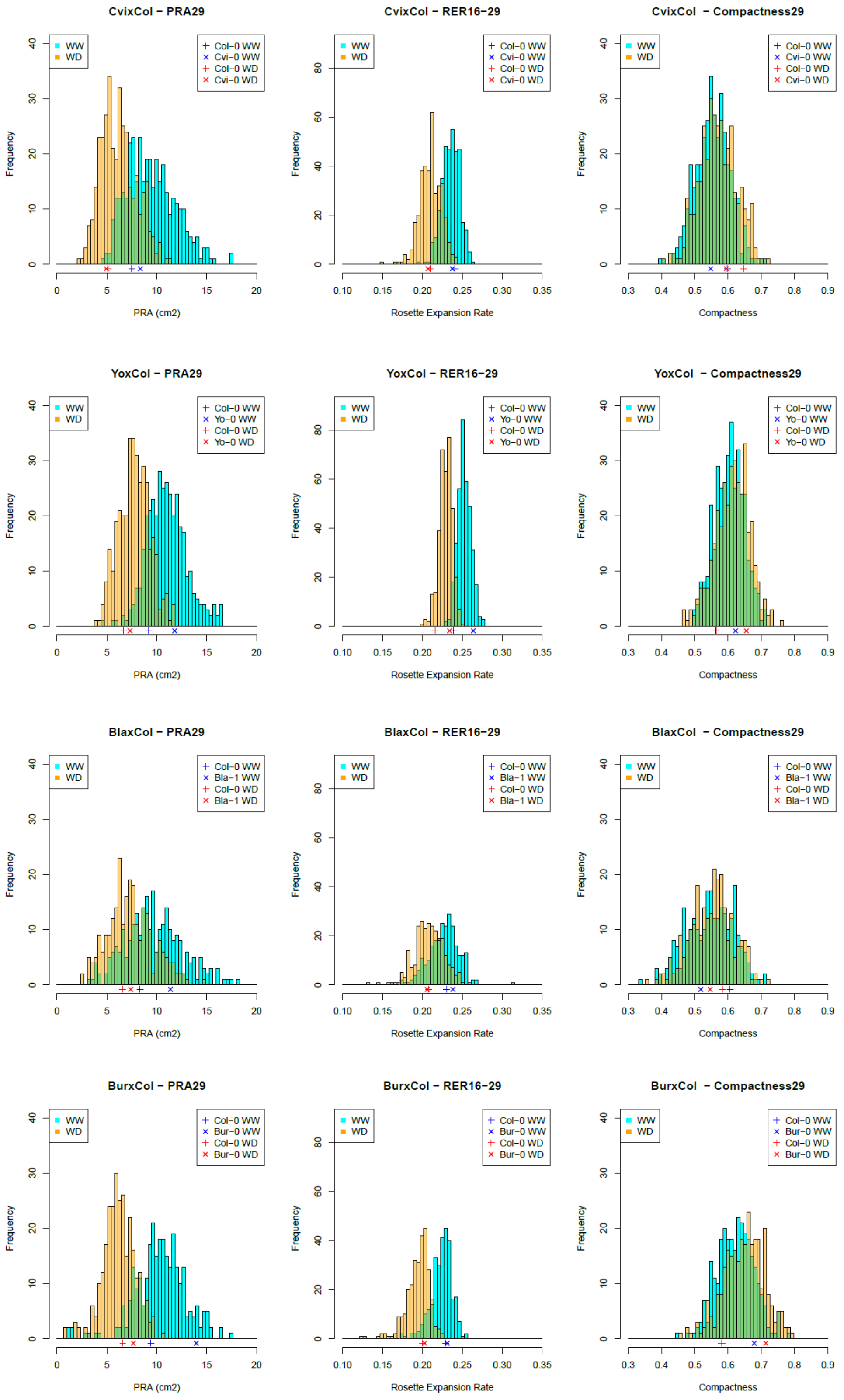

Supplementary Figure S1: Distributions of the mean phenotypic values across RILs

Frequency histograms showing the distributions of the PRA29, RER16-29 and Compactness29 traits within each of the four RIL sets in well watered (WW: blue) and water deficit (WD: orange) conditions. Phenotypic values for parental accessions from these specific experiments are indicated by blue (WW) and red (WD) ticks (see inset for legend) just above the x axes.
