# Supplementary material for: The complex genetic architecture of shoot growth natural variation in Arabidopsis thaliana
Source: PLoS Genet. 2019 Apr 22;15(4):e1007954. doi: 10.1371/journal.pgen.1007954 (PMC6476473; doi:10.1371/journal.pgen.1007954)
Supplement: S2 Fig — Distribution of the QTL effects (R2%) in the four RILs populations (CvixCol, BurxCol, BlaxCol, YoxCol), two conditions (WW and WD) and for the 3 traits considered in the analysis (PRA29, RER16-29, Compactness29). (PDF) [file pgen.1007954.s002.pdf]

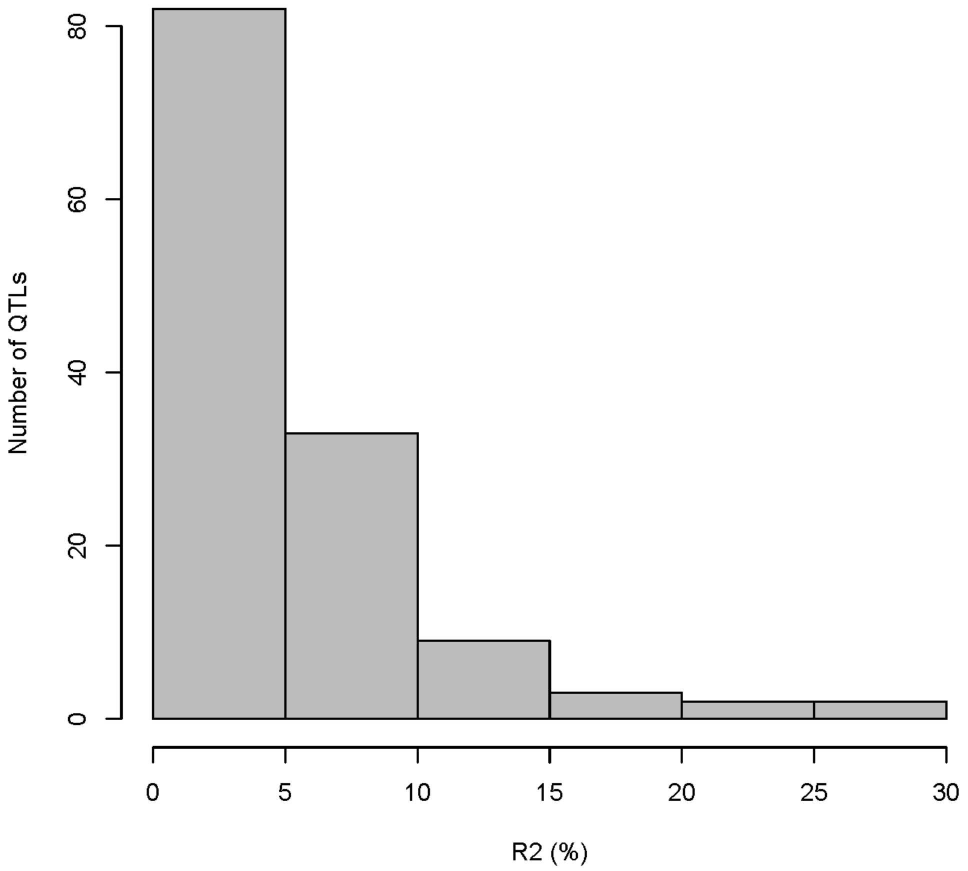

Supplementary Figure S2: Distribution of QTL effects

Distribution of the QTL effects (R2 %) in the four RILs populations (CvixCol, BurxCol, BlaxCol, YoxCol), two conditions (WW and WD) and for the 3 traits considered in the analysis (PRA29, RER16-29, Compactness29).
