# Supplementary material for: The complex genetic architecture of shoot growth natural variation in Arabidopsis thaliana
Source: PLoS Genet. 2019 Apr 22;15(4):e1007954. doi: 10.1371/journal.pgen.1007954 (PMC6476473; doi:10.1371/journal.pgen.1007954)
Supplement: S3 Fig — Heatmaps representing the LOD score of the interaction effects between all pairs of loci for 4 RIL sets, 3 traits, in 2 conditions. Each heatmap shows the pairwise interaction effects obtained in control condition WW (triangle above diagonal) and in water deficit condition WD (triangle below diagonal). The color scale (LOD score values) shared among all heatmaps is indicated on the right (note that it is different from the scale of Fig 4). Diagonal values are canceled and enlarged to exclude pairs of adjacent markers from the test. (PDF) [file pgen.1007954.s003.pdf]

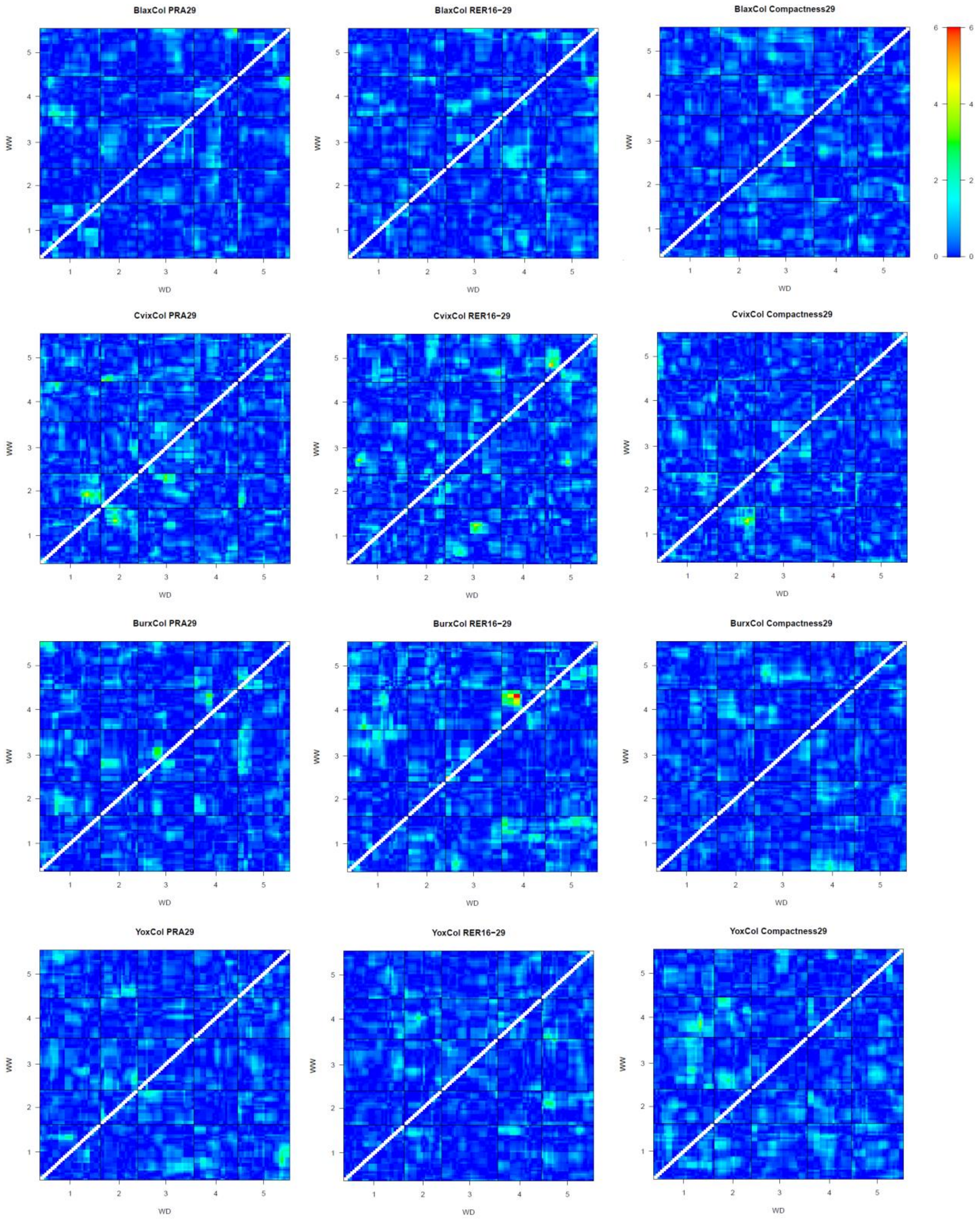

Supplementary Figure S3: 2D scans for epistasis

Heatmaps representing the LOD score of the interaction effects between all pairs of loci for 4 RIL sets, 3 traits, in 2 conditions. Each heatmap shows the pairwise interaction effects obtained in control condition WW (triangle above diagonal) and in water deficit condition WD (triangle below diagonal). The color scale (LOD score values) shared among all heatmaps is indicated on the right (note that it is different from the scale of @Figure 4). Diagonal values are canceled and enlarged to exclude pairs of adjacent markers from the test.
