# Supplementary material for: The complex genetic architecture of shoot growth natural variation in Arabidopsis thaliana
Source: PLoS Genet. 2019 Apr 22;15(4):e1007954. doi: 10.1371/journal.pgen.1007954 (PMC6476473; doi:10.1371/journal.pgen.1007954)
Supplement: S4 Fig — Same legend as Fig 5. Chromosome 3 shows no significant combined QTLs for RER16-29. (PDF) [file pgen.1007954.s004.pdf]

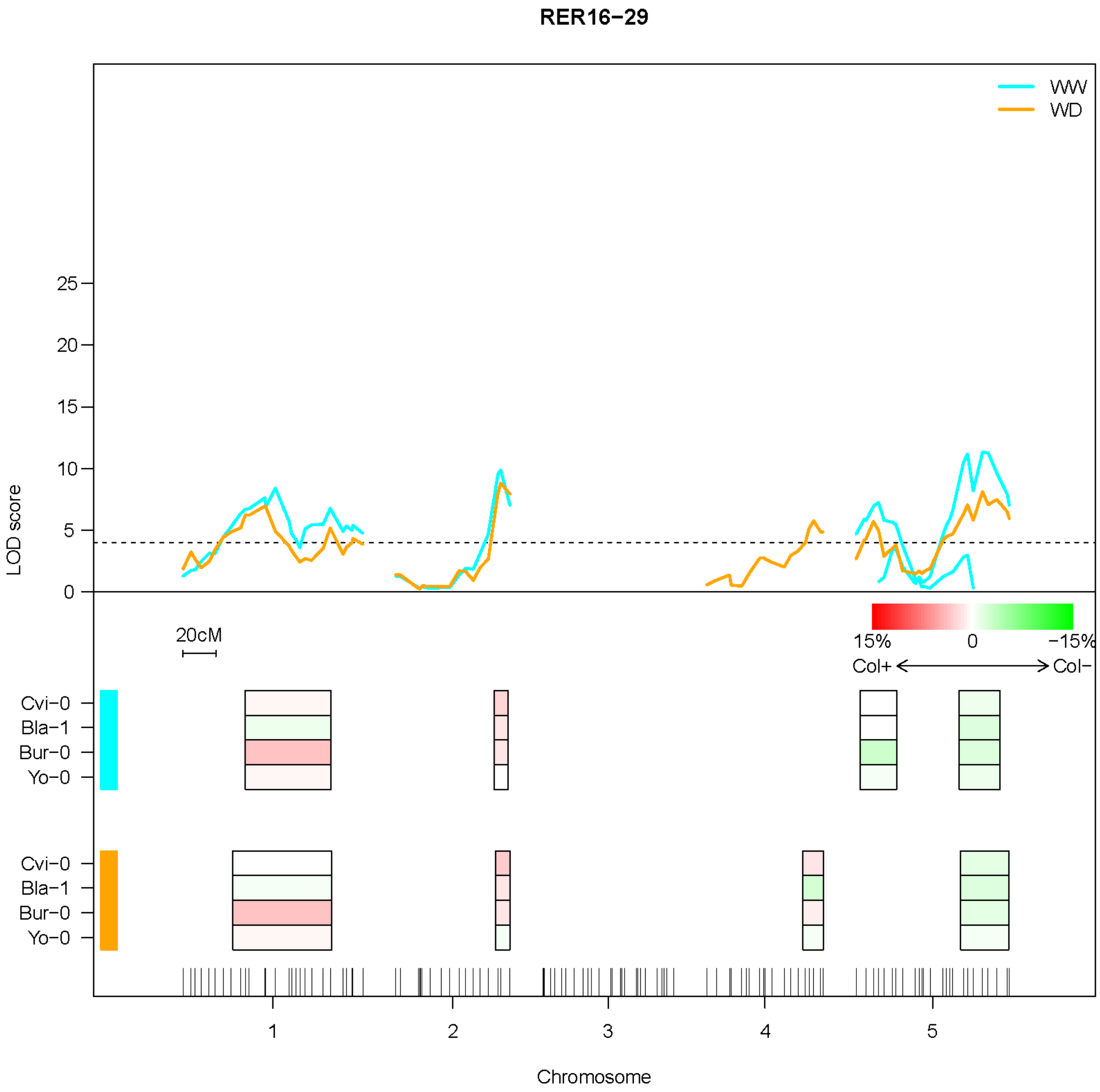

Supplementary Figure S4: Multi-cross QTL analysis for RER16-29

Same legend as @Figure 5.

Chromosome 3 shows no significant combined QTLs for RER16-29.
