# Supplementary figures and images for: The complex genetic architecture of shoot growth natural variation in Arabidopsis thaliana
Source: PLoS Genet. 2019 Apr 22;15(4):e1007954. doi: 10.1371/journal.pgen.1007954 (PMC6476473; doi:10.1371/journal.pgen.1007954)

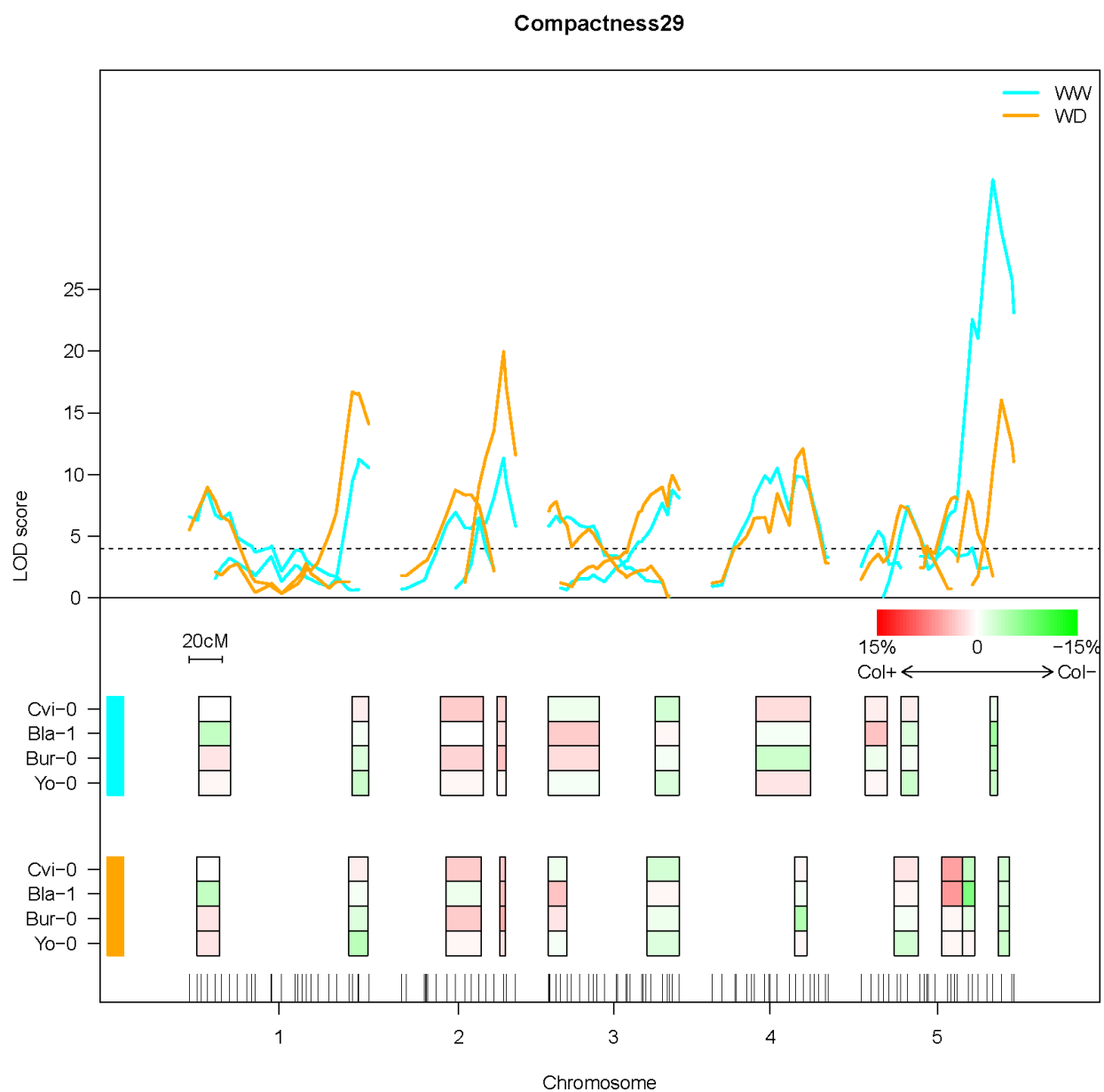

**Supplementary Figure S5: Multi-cross QTL analysis for Compactness29**

Same legend as @Figure 5.

Supplement: S5 Fig — Same legend as Fig 5. (PDF) [file pgen.1007954.s005.pdf]
