# Supplementary material for: The complex genetic architecture of shoot growth natural variation in Arabidopsis thaliana
Source: PLoS Genet. 2019 Apr 22;15(4):e1007954. doi: 10.1371/journal.pgen.1007954 (PMC6476473; doi:10.1371/journal.pgen.1007954)
Supplement: S6 Fig — Same legend as Fig 6. (PDF) [file pgen.1007954.s006.pdf]

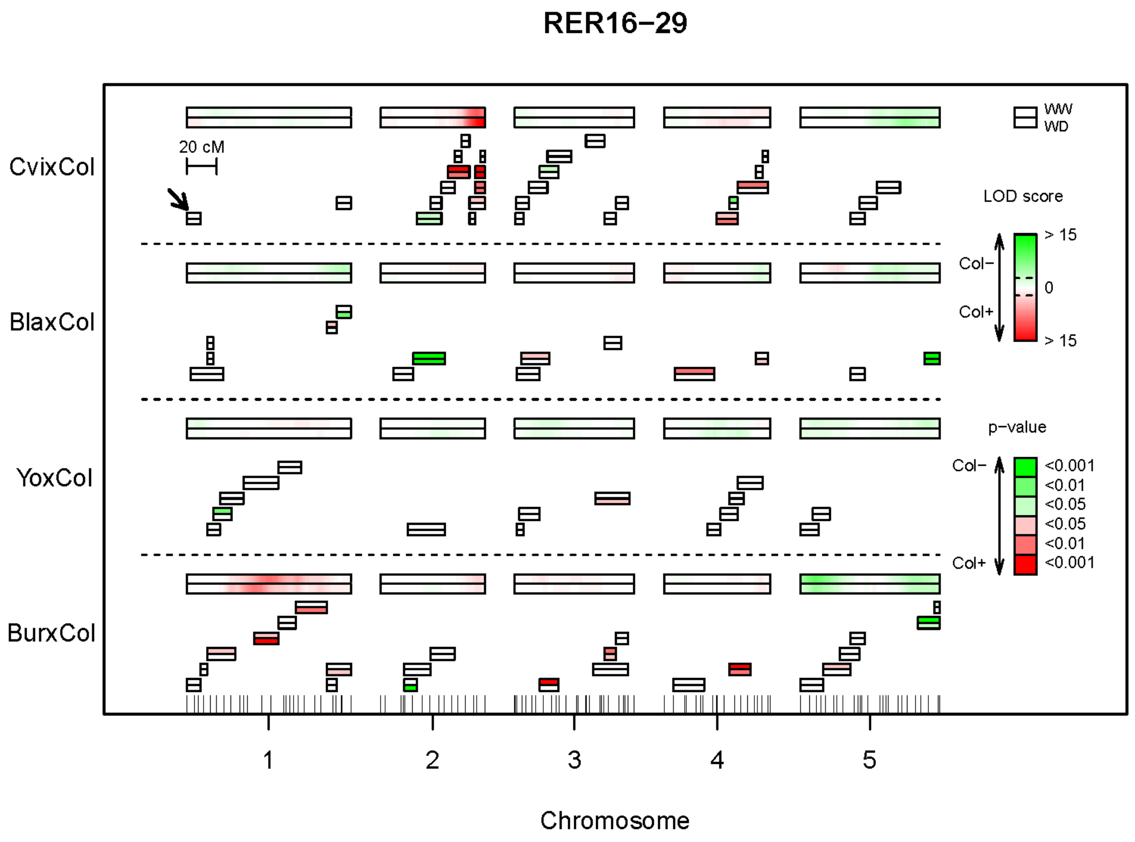

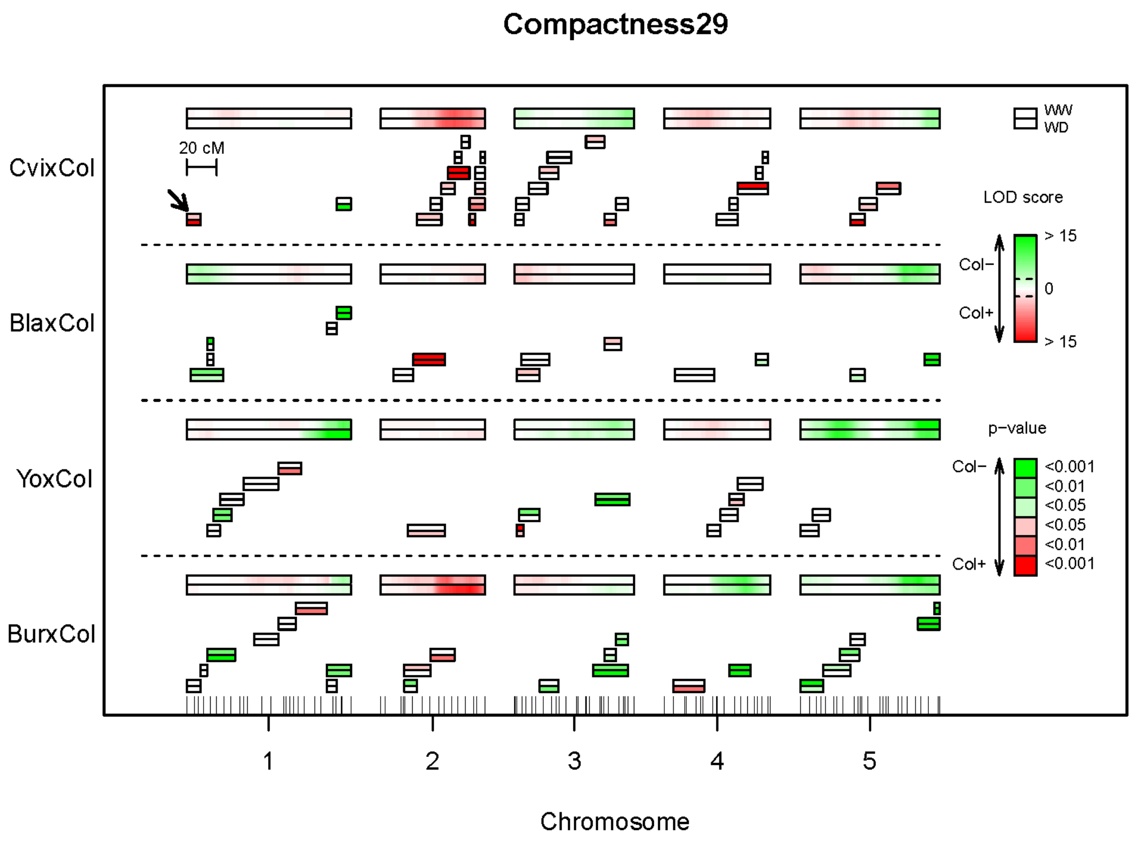

Supplementary Figure S6: Near isogenic lines-based validation of QTLs for RER16-29 and Compactness29. Same legend as @Figure 6
