# Supplementary material for: The complex genetic architecture of shoot growth natural variation in Arabidopsis thaliana
Source: PLoS Genet. 2019 Apr 22;15(4):e1007954. doi: 10.1371/journal.pgen.1007954 (PMC6476473; doi:10.1371/journal.pgen.1007954)
Supplement: S7 Fig — Same legend as Fig 7. (PDF) [file pgen.1007954.s007.pdf]

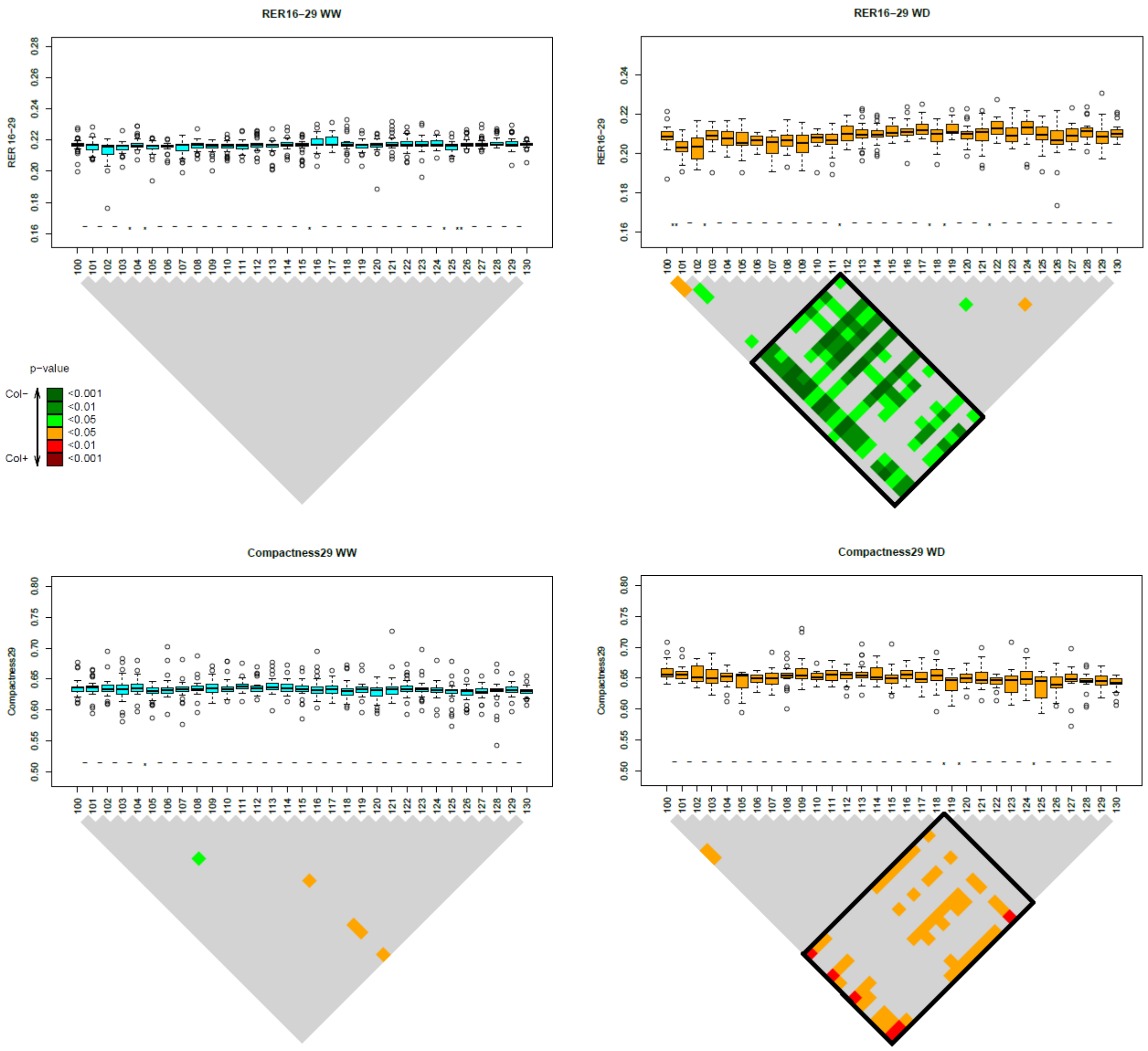


**Supplementary Figure S7: Dissection of a genomic region in CvixCol for RER16-29 and Compactness29 ('microStairs' approach)**

Same legend as @Figure 7
