# Supplementary material for: The complex genetic architecture of shoot growth natural variation in Arabidopsis thaliana
Source: PLoS Genet. 2019 Apr 22;15(4):e1007954. doi: 10.1371/journal.pgen.1007954 (PMC6476473; doi:10.1371/journal.pgen.1007954)
Supplement: S1 Table — Broad-sense heritabilities (h2) in the 4 RIL sets (BlaxCol, CvixCol, BurxCol and YoxCol) for PRA29, RER16-29 and Compactness29 in WW and WD conditions. H2 > 0.4 are highlighted in bold. Broad-sense heritabilities were calculated with the following equation h2 = Var(G)/Var(P) with Var(P) = Var(G)+Var(E) (PDF) [file pgen.1007954.s008.pdf]

Supplementary Table S1: Heritabilities of the observed phenotypes

Broad-sense heritabilities (h2) in the 4 RIL sets (BlaxCol, CvixCol, BurxCol and YoxCol) for PRA29, RER16-29 and Compactness29 in WW and WD conditions. H2 > 0.4 are highlighted in bold. Broad-sense heritabilities were calculated with the following equation h2 = Var(G)/Var(P) with Var(P)=Var(G)+Var(E)

	BlaxCol	CvixCol	BurxCol	YoxCol
PRA29 WW	0.23	**0.67**	**0.62**	**0.48**
PRA29 WD	0.12	**0.45**	**0.55**	0.26
RER16-29 WW	0.17	0.13	**0.66**	NA
RER16-29 WD	0.22	NA	**0.42**	NA
Compactness29 WW	**0.75**	**0.60**	**0.65**	**0.54**
Compactness29 WD	**0.50**	**0.40**	0.37	0.34
