# Supplementary material for: The complex genetic architecture of shoot growth natural variation in Arabidopsis thaliana
Source: PLoS Genet. 2019 Apr 22;15(4):e1007954. doi: 10.1371/journal.pgen.1007954 (PMC6476473; doi:10.1371/journal.pgen.1007954)
Supplement: S2 Table — For each RIL set, -log(p values) of main and interaction effects calculated by the analysis of variance (ANOVA) from the following model: Yijkl~μ+αi+βj+γk+δij+λjk+σik+εijkl. where Yijkl: phenotype; μ: mean; αi: effect of the condition; βj: effect of the experiment; γk: effect of the genotype; δij: effect of the interaction condition*experiment; λjk: effect of the interaction experiment*genotype; σik: effect of the interaction condition*genotype; εijkl: residuals. NS = Not Significant. (PDF) [file pgen.1007954.s009.pdf]

Supplementary Table S2: Analyses of variance for genotype, experiment and condition factors, and their interactions

For each RIL set, -log(p values) of main and interaction effects calculated by the analysis of variance (ANOVA) from the following model :

$$Y_{ijkl} \sim \mu + \alpha_i + \beta_j + \gamma_k + \delta_{ij} + \lambda_{jk} + \sigma_{ik} + \varepsilon_{ijkl}$$

where

Y_{ijkl}: phenotype; μ: mean; α_i: effect of the condition; β_j: effect of the experiment; γ_k: effect of the genotype; δ_{ij}: effect of the interaction condition*experiment; λ_{jk}: effect of the interaction experiment*genotype; σ_{ik}: effect of the interaction condition*genotype; ε_{ijkl}: residuals

NS = Not Significant

RIL set	Phenotype	Condition	Experiment	Genotype	Condition * Experiment	Experiment * Genotype	Condition * Genotype
CvixCol	PRA29	726,57	26,33	526,59	75,81	51,37	16,87
CvixCol	Compactness29	57,54	212,95	378,58	50,64	13,01	NS
CvixCol	RER16-29	555,13	499,28	138,67	69,48	8,13	3,06
YoxCol	PRA29	450,11	27,20	185,91	20,90	47,28	3,30
YoxCol	Compactness29	12,03	96,39	151,05	25,42	NS	NS
YoxCol	RER16-29	482,02	355,28	84,52	8,39	6,57	NS
BlaxCol	PRA29	211,83	10,96	212,99	NS	184,35	9,44
BlaxCol	Compactness29	NS	4,27	183,33	NS	4,85	4,96
BlaxCol	RER16-29	161,24	47,86	112,12	NS	73,21	3,69
BurxCol	PRA29	402,90	14,73	168,74	7,37	4,92	11,24
BurxCol	Compactness29	34,46	8,21	91,88	NS	NS	NS
BurxCol	RER16-29	407,91	22,25	149,56	NS	11,60	13,24
