# Supplementary material for: The complex genetic architecture of shoot growth natural variation in Arabidopsis thaliana
Source: PLoS Genet. 2019 Apr 22;15(4):e1007954. doi: 10.1371/journal.pgen.1007954 (PMC6476473; doi:10.1371/journal.pgen.1007954)
Supplement: S3 Table — For each RIL set, trait and condition (or GxE interaction term: 'inter'): every independent QTL peak is a row in the table, indicating its localisation (chromosome and position on the genetic map), its significance (maximum LOD score) and its effect (direction of the allelic effect: 'sign' indicates the sign of the allelic effect estimated as [Col–Xxx], where Xxx is the alternate parental allele; R2%). Independent QTLs peaks are considered when the LOD score curve returns below threshold between 2 peaks, to ensure the loci are not too genetically linked. (PDF) [file pgen.1007954.s010.pdf]

Supplementary Table S3: Mapped QTL parameters across RIL sets, traits and conditions

For each RIL set, trait and condition (or GxE interaction term: 'inter'): every independent QTL peak is a row in the table, indicating its localisation (chromosome and position on the genetic map), its significance (maximum LOD score) and its effect (direction of the allelic effect: 'sign' indicates the sign of the allelic effect estimated as [Col – Xxx], where Xxx is the alternate parental allele; R2 %). Independent QTLs peaks are considered when the LOD score curve returns below threshold between 2 peaks, to ensure the loci are not too genetically linked.

RIL set	Phenotype	Condition	Chromosome	Position (cM)	LOD score	sign	R2 (%)
BurxCol	PRA29	WD	1	5	4,1	-1	1
BurxCol	PRA29	WD	1	50	11,2	1	11
BurxCol	PRA29	WD	5	93	2,6	-1	3
BurxCol	PRA29	WW	1	11	9,1	-1	4
BurxCol	PRA29	WW	1	50	19,8	1	12
BurxCol	PRA29	WW	4	0	8,3	-1	9
BurxCol	PRA29	WW	5	14	2,8	-1	2
BurxCol	PRA29	WW	5	93	3,2	-1	3
BurxCol	PRA29	inter	1	20	9	-1	7
BurxCol	PRA29	inter	1	50	6,6	1	4
BurxCol	PRA29	inter	2	70	3,9	1	3
BurxCol	PRA29	inter	4	0	5,3	-1	6
BurxCol	RER16-29	WD	1	50	9,3	1	11
BurxCol	RER16-29	WD	1	74	3,1	1	4
BurxCol	RER16-29	WD	1	90	2,9	1	8
BurxCol	RER16-29	WD	5	11	9,3	-1	6
BurxCol	RER16-29	WD	5	86	4,3	-1	3
BurxCol	RER16-29	WW	1	56	12,4	1	9
BurxCol	RER16-29	WW	2	64	2,9	1	2
BurxCol	RER16-29	WW	5	11	16	-1	12
BurxCol	RER16-29	WW	5	80	8	-1	4
BurxCol	RER16-29	inter	1	25	3,2	-1	5
BurxCol	Compactness29	WD	1	110	4,1	-1	3
BurxCol	Compactness29	WD	2	56	16,4	1	11
BurxCol	Compactness29	WD	4	56	12	-1	8
BurxCol	Compactness29	WD	5	86	7,4	-1	3
BurxCol	Compactness29	WW	1	104	7,2	-1	3
BurxCol	Compactness29	WW	1	50	2,8	1	1
BurxCol	Compactness29	WW	1	65	2,9	1	1
BurxCol	Compactness29	WW	2	47	14,6	1	11
BurxCol	Compactness29	WW	3	14	3,7	1	3
BurxCol	Compactness29	WW	4	56	13,7	-1	6
BurxCol	Compactness29	WW	5	6	4	-1	1
BurxCol	Compactness29	WW	5	80	17	-1	8
BurxCol	Compactness29	inter	3	59	2,7	1	4
YoxCol	PRA29	WD	1	20	4	-1	4

YoxCol	PRA29	WD	1	56	3,6	-1	5
YoxCol	PRA29	WD	2	33	4,2	-1	3
YoxCol	PRA29	WD	4	21	2,7	-1	3
YoxCol	PRA29	WD	4	47	2,9	-1	4
YoxCol	PRA29	WD	4	60	2,5	-1	4
YoxCol	PRA29	WD	5	80	11,6	-1	9
YoxCol	PRA29	WD	5	6	3,9	1	3
YoxCol	PRA29	WW	1	16	3,1	-1	4
YoxCol	PRA29	WW	1	35	5,7	-1	7
YoxCol	PRA29	WW	2	39	3	-1	4
YoxCol	PRA29	WW	4	15	6,2	-1	4
YoxCol	PRA29	WW	5	77	16,8	-1	12
YoxCol	PRA29	WW	5	11	9,6	1	6
YoxCol	PRA29	inter	4	0	3,6	-1	3
YoxCol	PRA29	inter	4	71	2,6	1	3
YoxCol	PRA29	inter	5	68	5,6	-1	4
YoxCol	PRA29	inter	5	11	4,9	1	4
YoxCol	RER16-29	WD	4	35	3,6	-1	4
YoxCol	RER16-29	WD	4	51	2,9	-1	5
YoxCol	RER16-29	WD	5	6	3	-1	4
YoxCol	RER16-29	WD	5	65	5,3	-1	5
YoxCol	RER16-29	WW	1	11	3,6	-1	3
YoxCol	RER16-29	WW	3	19	2,6	-1	4
YoxCol	RER16-29	WW	5	14	3,2	-1	4
YoxCol	RER16-29	WW	5	71	9,7	-1	11
YoxCol	RER16-29	inter	4	65	4,9	1	6
YoxCol	RER16-29	inter	5	92	3	-1	3
YoxCol	Compactness29	WD	1	104	27,6	-1	18
YoxCol	Compactness29	WD	1	11	2,4	1	5
YoxCol	Compactness29	WD	2	64	4,9	1	5
YoxCol	Compactness29	WD	3	69	6,6	-1	5
YoxCol	Compactness29	WD	4	32	4,7	1	3
YoxCol	Compactness29	WD	5	24	18,2	-1	10
YoxCol	Compactness29	WD	5	86	15,8	-1	13
YoxCol	Compactness29	WW	1	35	2,8	-1	1
YoxCol	Compactness29	WW	1	104	14,6	-1	10
YoxCol	Compactness29	WW	1	11	2,8	1	3
YoxCol	Compactness29	WW	3	69	8,2	-1	7
YoxCol	Compactness29	WW	4	35	6	1	3
YoxCol	Compactness29	WW	4	71	3,3	1	3
YoxCol	Compactness29	WW	5	28	16,8	-1	10
YoxCol	Compactness29	WW	5	86	24,7	-1	19
YoxCol	Compactness29	inter	1	85	5,1	1	7
YoxCol	Compactness29	inter	2	64	2,6	-1	3
BlaxCol	PRA29	WD	1	20	3,8	-1	6
BlaxCol	PRA29	WW	1	16	4,5	-1	7
BlaxCol	PRA29	WW	1	110	5,8	-1	6
BlaxCol	PRA29	inter	1	110	3,5	-1	6
BlaxCol	RER16-29	WD	3	69	2,8	1	4
BlaxCol	RER16-29	WD	4	65	2,5	-1	4
BlaxCol	RER16-29	WW	1	104	2,7	-1	3
BlaxCol	RER16-29	WW	3	76	3,5	1	4

BlaxCol	RER16-29	WW	5	65	2,5	-1	5
BlaxCol	RER16-29	WW	5	80	3,2	-1	4
BlaxCol	Compactness29	WD	1	11	6,8	-1	8
BlaxCol	Compactness29	WD	2	64	8,2	1	6
BlaxCol	Compactness29	WD	3	0	9,6	1	9
BlaxCol	Compactness29	WD	5	77	12,2	-1	10
BlaxCol	Compactness29	WW	1	11	6,3	-1	8
BlaxCol	Compactness29	WW	1	74	2,7	1	5
BlaxCol	Compactness29	WW	2	64	3,8	1	2
BlaxCol	Compactness29	WW	3	0	5,5	1	4
BlaxCol	Compactness29	WW	5	77	22,4	-1	20
BlaxCol	Compactness29	WW	5	11	5,6	1	4
CvixCol	PRA29	WD	1	20	4,5	-1	4
CvixCol	PRA29	WD	2	70	29,6	1	30
CvixCol	PRA29	WD	4	47	2,6	-1	1
CvixCol	PRA29	WD	5	86	6,3	-1	4
CvixCol	PRA29	WW	1	16	9,4	-1	7
CvixCol	PRA29	WW	1	104	3	-1	4
CvixCol	PRA29	WW	2	64	27,3	1	24
CvixCol	PRA29	WW	4	47	6,9	-1	3
CvixCol	PRA29	WW	5	86	9,4	-1	4
CvixCol	PRA29	inter	1	0	7,5	-1	8
CvixCol	PRA29	inter	2	42	3,4	1	4
CvixCol	PRA29	inter	4	47	4,9	-1	4
CvixCol	RER16-29	WD	2	70	32	1	27
CvixCol	RER16-29	WD	5	71	7	-1	4
CvixCol	RER16-29	WW	2	64	23,3	1	22
CvixCol	RER16-29	WW	5	80	4,8	-1	4
CvixCol	RER16-29	inter	1	5	2,7	-1	3
CvixCol	Compactness29	WD	2	47	14,2	1	8
CvixCol	Compactness29	WD	3	80	9,4	-1	7
CvixCol	Compactness29	WD	4	21	2,9	1	2
CvixCol	Compactness29	WD	5	86	7,8	-1	4
CvixCol	Compactness29	WD	5	36	2,5	1	1
CvixCol	Compactness29	WD	5	55	2,6	1	2
CvixCol	Compactness29	WW	1	29	2,5	1	2
CvixCol	Compactness29	WW	2	47	11,6	1	8
CvixCol	Compactness29	WW	3	5	3	-1	2
CvixCol	Compactness29	WW	3	80	9,4	-1	7
CvixCol	Compactness29	WW	4	15	3,5	1	3
CvixCol	Compactness29	WW	4	32	2,8	1	4
CvixCol	Compactness29	WW	5	92	8,4	-1	4
CvixCol	Compactness29	WW	5	55	2,6	1	2
