# Supplementary material for: The complex genetic architecture of shoot growth natural variation in Arabidopsis thaliana
Source: PLoS Genet. 2019 Apr 22;15(4):e1007954. doi: 10.1371/journal.pgen.1007954 (PMC6476473; doi:10.1371/journal.pgen.1007954)
Supplement: S4 Table — The genotypes of the 31 lines selected for the microStairs experiment are indicated along this 3Mb region of the top of chromosome 1 with markers localized on the physical map (kb). (PDF) [file pgen.1007954.s011.pdf]

Supplementary Table S4: microStairs; genotypic description of recombinant lines

The genotypes of the 31 lines selected for the microStairs experiment are indicated along this 3Mb region of the top of chromosome 1 with markers localised on the physical map (kb).

				recomb. name	8HV294100	8HV294101	8HV294102	8HV294103	8HV294104	8HV294105	8HV294106	8HV294107	8HV294108	8HV294109	8HV294110	8HV294111	8HV294112	8HV294113	8HV294114	8HV294115	8HV294116	8HV294117	8HV294118	8HV294119	8HV294120	8HV294121	8HV294122	8HV294123	8HV294124	8HV294125	8HV294126	8HV294127	8HV294128	8HV294129	8HV294130	
**Marker name**	**Marker pos. (kb, Chrom 1)**	**Min Bin size (kb)**	**Max Bin size (kb)**	**Rough position**	**100**	**101**	**102**	**103**	**104**	**105**	**106**	**107**	**108**	**109**	**110**	**111**	**112**	**113**	**114**	**115**	**116**	**117**	**118**	**119**	**120**	**121**	**122**	**123**	**124**	**125**	**126**	**127**	**128**	**129**	**130**	**< Names on Figure 7 & S7**
MSAT1.00008	8 639			0	Col	Cvi	Cvi	Cvi	Cvi	Cvi	Cvi	Cvi	Cvi	Cvi	Cvi	Cvi	Cvi	Cvi	Cvi	Cvi	Cvi	Cvi	Cvi	Cvi	Cvi	Cvi	Cvi	Cvi	Cvi	Cvi	Cvi	Cvi	Cvi	Cvi	Cvi	
HRM1.00084.F1b/R1b	84 908	76 269	161 913	0,1	Col	Cvi	Cvi	Cvi	Cvi	Cvi	Cvi	Cvi	Cvi	Cvi	Cvi	Cvi	Cvi	Cvi	Cvi	Cvi	Cvi	Cvi	Cvi	Cvi	Cvi	Cvi	Cvi	Cvi	Cvi	Cvi	Cvi	Cvi	Cvi	Cvi	Cvi	
IND1.00171	170 552	85 644	213 230	0,2	Col	Col	Cvi	Cvi	Cvi	Cvi	Cvi	Cvi	Cvi	Cvi	Cvi	Cvi	Cvi	Cvi	Cvi	Cvi	Cvi	Cvi	Cvi	Cvi	Cvi	Cvi	Cvi	Cvi	Cvi	Cvi	Cvi	Cvi	Cvi	Cvi	Cvi	
IND1.00298	298 138	127 586	256 791	0,3	Col	Col	Col	Cvi	Cvi	Cvi	Cvi	Cvi	Cvi	Cvi	Cvi	Cvi	Cvi	Cvi	Cvi	Cvi	Cvi	Cvi	Cvi	Cvi	Cvi	Cvi	Cvi	Cvi	Cvi	Cvi	Cvi	Cvi	Cvi	Cvi	Cvi	
T7I23	427 343	129 205	215 153	0,4	Col	Col	Col	Col	Cvi	Cvi	Cvi	Cvi	Cvi	Cvi	Cvi	Cvi	Cvi	Cvi	Cvi	Cvi	Cvi	Cvi	Cvi	Cvi	Cvi	Cvi	Cvi	Cvi	Cvi	Cvi	Cvi	Cvi	Cvi	Cvi	Cvi	
IND1.00513	513 291	85 948	189 488	0,5	Col	Col	Col	Col	Col	Cvi	Cvi	Cvi	Cvi	Cvi	Cvi	Cvi	Cvi	Cvi	Cvi	Cvi	Cvi	Cvi	Cvi	Cvi	Cvi	Cvi	Cvi	Cvi	Cvi	Cvi	Cvi	Cvi	Cvi	Cvi	Cvi	
HRM1.000616.F2b/R2	616 831	103 540	186 219	0,6	Col	Col	Col	Col	Col	Col	Cvi	Cvi	Cvi	Cvi	Cvi	Cvi	Cvi	Cvi	Cvi	Cvi	Cvi	Cvi	Cvi	Cvi	Cvi	Cvi	Cvi	Cvi	Cvi	Cvi	Cvi	Cvi	Cvi	Cvi	Cvi	
HRM1.00699	699 510	82 679	189 317	0,7	Col	Col	Col	Col	Col	Col	Col	Cvi	Cvi	Cvi	Cvi	Cvi	Cvi	Cvi	Cvi	Cvi	Cvi	Cvi	Cvi	Cvi	Cvi	Cvi	Cvi	Cvi	Cvi	Cvi	Cvi	Cvi	Cvi	Cvi	Cvi	
HRM1.00806	806 148	106 638	199 053	0,8	Col	Col	Col	Col	Col	Col	Col	Col	Cvi	Cvi	Cvi	Cvi	Cvi	Cvi	Cvi	Cvi	Cvi	Cvi	Cvi	Cvi	Cvi	Cvi	Cvi	Cvi	Cvi	Cvi	Cvi	Cvi	Cvi	Cvi	Cvi	
IND1.00899	898 563	92 415	200 402	0,9	Col	Col	Col	Col	Col	Col	Col	Col	Col	Cvi	Cvi	Cvi	Cvi	Cvi	Cvi	Cvi	Cvi	Cvi	Cvi	Cvi	Cvi	Cvi	Cvi	Cvi	Cvi	Cvi	Cvi	Cvi	Cvi	Cvi	Cvi	
HRM1.01007	1 006 550	107 987	172 012	1	Col	Col	Col	Col	Col	Col	Col	Col	Col	Col	Cvi	Cvi	Cvi	Cvi	Cvi	Cvi	Cvi	Cvi	Cvi	Cvi	Cvi	Cvi	Cvi	Cvi	Cvi	Cvi	Cvi	Cvi	Cvi	Cvi	Cvi	
F20D22#2	1 070 575	64 025	208 869	1,1	Col	Col	Col	Col	Col	Col	Col	Col	Col	Col	Col	Cvi	Cvi	Cvi	Cvi	Cvi	Cvi	Cvi	Cvi	Cvi	Cvi	Cvi	Cvi	Cvi	Cvi	Cvi	Cvi	Cvi	Cvi	Cvi	Cvi	
IND1.01215bR	1 215 419	144 844	221 028	1,2	Col	Col	Col	Col	Col	Col	Col	Col	Col	Col	Col	Col	Cvi	Cvi	Cvi	Cvi	Cvi	Cvi	Cvi	Cvi	Cvi	Cvi	Cvi	Cvi	Cvi	Cvi	Cvi	Cvi	Cvi	Cvi	Cvi	
HRM1.01292	1 291 603	76 184	203 857	1,3	Col	Col	Col	Col	Col	Col	Col	Col	Col	Col	Col	Col	Col	Cvi	Cvi	Cvi	Cvi	Cvi	Cvi	Cvi	Cvi	Cvi	Cvi	Cvi	Cvi	Cvi	Cvi	Cvi	Cvi	Cvi	Cvi	
IND1.01419	1 419 276	127 673	203 953	1,4	Col	Col	Col	Col	Col	Col	Col	Col	Col	Col	Col	Col	Col	Col	Cvi	Cvi	Cvi	Cvi	Cvi	Cvi	Cvi	Cvi	Cvi	Cvi	Cvi	Cvi	Cvi	Cvi	Cvi	Cvi	Cvi	
EMIND1.01496	1 495 556	76 280	184 549	1,5	Col	Col	Col	Col	Col	Col	Col	Col	Col	Col	Col	Col	Col	Col	Col	Cvi	Cvi	Cvi	Cvi	Cvi	Cvi	Cvi	Cvi	Cvi	Cvi	Cvi	Cvi	Cvi	Cvi	Cvi	Cvi	
EMIND1.01604	1 603 825	108 269	208 045	1,6	Col	Col	Col	Col	Col	Col	Col	Col	Col	Col	Col	Col	Col	Col	Col	Col	Cvi	Cvi	Cvi	Cvi	Cvi	Cvi	Cvi	Cvi	Cvi	Cvi	Cvi	Cvi	Cvi	Cvi	Cvi	
HRM1.01704	1 703 601	99 776	197 220	1,7	Col	Col	Col	Col	Col	Col	Col	Col	Col	Col	Col	Col	Col	Col	Col	Col	Col	Cvi	Cvi	Cvi	Cvi	Cvi	Cvi	Cvi	Cvi	Cvi	Cvi	Cvi	Cvi	Cvi	Cvi	
HRM1.01801	1 801 045	97 444	218 397	1,8	Col	Col	Col	Col	Col	Col	Col	Col	Col	Col	Col	Col	Col	Col	Col	Col	Col	Col	Cvi	Cvi	Cvi	Cvi	Cvi	Cvi	Cvi	Cvi	Cvi	Cvi	Cvi	Cvi	Cvi	
HRM1.01922	1 921 998	120 953	225 955	1,9	Col	Col	Col	Col	Col	Col	Col	Col	Col	Col	Col	Col	Col	Col	Col	Col	Col	Col	Col	Cvi	Cvi	Cvi	Cvi	Cvi	Cvi	Cvi	Cvi	Cvi	Cvi	Cvi	Cvi	
HRM1.02027 F1/R1b	2 027 000	105 002	183 086	2	Col	Col	Col	Col	Col	Col	Col	Col	Col	Col	Col	Col	Col	Col	Col	Col	Col	Col	Col	Col	Cvi	Cvi	Cvi	Cvi	Cvi	Cvi	Cvi	Cvi	Cvi	Cvi	Cvi	
MSAT1.02105	2 105 084	78 084	169 604	2,1	Col	Col	Col	Col	Col	Col	Col	Col	Col	Col	Col	Col	Col	Col	Col	Col	Col	Col	Col	Col	Col	Cvi	Cvi	Cvi	Cvi	Cvi	Cvi	Cvi	Cvi	Cvi	Cvi	
HRM1.02197	2 196 604	91 520	179 307	2,2	Col	Col	Col	Col	Col	Col	Col	Col	Col	Col	Col	Col	Col	Col	Col	Col	Col	Col	Col	Col	Col	Col	Cvi	Cvi	Cvi	Cvi	Cvi	Cvi	Cvi	Cvi	Cvi	
HRM1.02284	2 284 391	87 787	209 037	2,3	Col	Col	Col	Col	Col	Col	Col	Col	Col	Col	Col	Col	Col	Col	Col	Col	Col	Col	Col	Col	Col	Col	Col	Cvi	Cvi	Cvi	Cvi	Cvi	Cvi	Cvi	Cvi	
AT1G07790_HRM2_FW	2 405 641	121 250	225 462	2,4	Col	Col	Col	Col	Col	Col	Col	Col	Col	Col	Col	Col	Col	Col	Col	Col	Col	Col	Col	Col	Col	Col	Col	Col	Cvi	Cvi	Cvi	Cvi	Cvi	Cvi	Cvi	
HRM1.02509	2 509 853	104 212	181 070	2,5	Col	Col	Col	Col	Col	Col	Col	Col	Col	Col	Col	Col	Col	Col	Col	Col	Col	Col	Col	Col	Col	Col	Col	Col	Col	Cvi	Cvi	Cvi	Cvi	Cvi	Cvi	
HRM1.02586.F2/R2	2 586 711	76 858	200 432	2,6	Col	Col	Col	Col	Col	Col	Col	Col	Col	Col	Col	Col	Col	Col	Col	Col	Col	Col	Col	Col	Col	Col	Col	Col	Col	Col	Cvi	Cvi	Cvi	Cvi	Cvi	
HRM1.02710	2 710 285	123 574	223 966	2,7	Col	Col	Col	Col	Col	Col	Col	Col	Col	Col	Col	Col	Col	Col	Col	Col	Col	Col	Col	Col	Col	Col	Col	Col	Col	Col	Col	Cvi	Cvi	Cvi	Cvi	
HRM1.02811b	2 810 677	100 392	204 895	2,8	Col	Col	Col	Col	Col	Col	Col	Col	Col	Col	Col	Col	Col	Col	Col	Col	Col	Col	Col	Col	Col	Col	Col	Col	Col	Col	Col	Col	Cvi	Cvi	Cvi	
HRM1.02915	2 915 180	104 503	198 440	2,9	Col	Col	Col	Col	Col	Col	Col	Col	Col	Col	Col	Col	Col	Col	Col	Col	Col	Col	Col	Col	Col	Col	Col	Col	Col	Col	Col	Col	Col	Cvi	Cvi	
HRM1.03009	3 009 117	93 937	181 820	3	Col	Col	Col	Col	Col	Col	Col	Col	Col	Col	Col	Col	Col	Col	Col	Col	Col	Col	Col	Col	Col	Col	Col	Col	Col	Col	Col	Col	Col	Col	Cvi	
HRM1.03097	3 097 000	87 883	130 943	3,1	Col	Col	Col	Col	Col	Col	Col	Col	Col	Col	Col	Col	Col	Col	Col	Col	Col	Col	Col	Col	Col	Col	Col	Col	Col	Col	Col	Col	Col	Col	Cvi	
mHIND1.03140	3 140 060	43 060			Cvi	Cvi	Cvi	Cvi	Cvi	Cvi	Cvi	Cvi	Cvi	Cvi	Cvi	Cvi	Cvi	Cvi	Cvi	Cvi	Cvi	Cvi	Cvi	Cvi	Cvi	Cvi	Cvi	Cvi	Cvi	Cvi	Cvi	Cvi	Cvi	Cvi	Cvi	
