# Supplementary material for: The complex genetic architecture of shoot growth natural variation in Arabidopsis thaliana
Source: PLoS Genet. 2019 Apr 22;15(4):e1007954. doi: 10.1371/journal.pgen.1007954 (PMC6476473; doi:10.1371/journal.pgen.1007954)
Supplement: S5 Table — This table lists all known polymorphisms between Col-0 and Cvi-0 that can potentially affect gene function (according to 1001Genomes data) within PRA29-significant bins only: in the selected physical interval, genes with more than 3 non-synonymous SNP or a high-impact polymorphism are highlighted in yellow and orange respectively. CRY2 candidate gene is highlighted in red. (PDF) [file pgen.1007954.s012.pdf]

Supplementary Table S5: microStairs; polymorphisms segregating in different bins

This table lists all known polymorphisms between Col-0 and Cvi-0 that can potentially affect gene function (according to 1001Genomes data) within PRA29-significant bins only: in the selected physical interval, genes with more than 3 non-synonymous SNP or a high-impact polymorphism are highlighted in yellow and orange respectively. CRY2 candidate gene is highlighted in red.

intervals microStairs	gene id	region	funct_consequence	chr	pos	Col allele	Cvi allele	Gene generic name
101/102	AT1G01220	exonic	nonsynonymous SNV	Chr1	95386	A	C	
101/102	AT1G01260	exonic	nonsynonymous SNV	Chr1	111074	T	G	
101/102	AT1G01260	exonic	nonsynonymous SNV	Chr1	111321	G	C	
101/102	AT1G01300	exonic	nonsynonymous SNV	Chr1	117329	G	C	Eukaryotic aspartyl protease family protein
101/102	AT1G01300	exonic	nonsynonymous SNV	Chr1	117331	C	A	
101/102	AT1G01300	exonic	nonsynonymous SNV	Chr1	117375	A	G	
101/102	AT1G01300	exonic	nonsynonymous SNV	Chr1	117675	G	T	
101/102	AT1G01300	exonic	nonsynonymous SNV	Chr1	118006	C	G	
101/102	AT1G01310	exonic	nonsynonymous SNV	Chr1	120661	C	G	
101/102	AT1G01320	exonic	nonsynonymous SNV	Chr1	123313	G	C	REC1, REDUCED CHLOROPLAST COVERAGE
101/102	AT1G01320	exonic	nonsynonymous SNV	Chr1	127093	T	C	
101/102	AT1G01320	exonic	nonsynonymous SNV	Chr1	127891	C	T	
101/102	AT1G01320	exonic	nonsynonymous SNV	Chr1	129003	T	G	
101/102	AT1G01340	exonic	nonsynonymous SNV	Chr1	134470	T	G	
101/102	AT1G01350	exonic	nonsynonymous SNV	Chr1	137008	C	G	
101/102	AT1G01350	exonic	nonsynonymous SNV	Chr1	137115	C	T	
101/102	AT1G01355	exonic	nonsynonymous SNV	Chr1	138608	C	G	
101/102	AT1G01370	exonic	nonsynonymous SNV	Chr1	144331	C	A	
101/102	AT1G01390	exonic	nonsynonymous SNV	Chr1	148357	T	C	
101/102	AT1G01390	exonic	nonsynonymous SNV	Chr1	149437	T	G	
101/102	AT1G01400	exonic	nonsynonymous SNV	Chr1	151491	C	T	hypothetical protein
101/102	AT1G01400	exonic	nonsynonymous SNV	Chr1	151494	C	T	
101/102	AT1G01400	exonic	nonsynonymous SNV	Chr1	151617	A	G	
101/102	AT1G01400	exonic	nonsynonymous SNV	Chr1	151942	A	C	
101/102	AT1G01410	exonic	frameshift deletion	Chr1	154016	AT	A	APUM22, PUM22, PUMILIO 22

101/102	AT1G01410	exonic	nonsynonymous SNV	Chr1	153232	T	A	
101/102	AT1G01420	exonic	nonsynonymous SNV	Chr1	154679	A	C	UDP-GLUCOSYL TRANSFERASE 72B3
101/102	AT1G01420	exonic	nonsynonymous SNV	Chr1	155442	T	G	
101/102	AT1G01420	exonic	nonsynonymous SNV	Chr1	155626	T	A	
101/102	AT1G01420	exonic	nonsynonymous SNV	Chr1	155678	C	A	
101/102	AT1G01420	exonic	nonsynonymous SNV	Chr1	155809	A	G	
101/102	AT1G01420	exonic	nonsynonymous SNV	Chr1	155921	G	C	
101/102	AT1G01420	exonic	nonsynonymous SNV	Chr1	155984	C	A	
101/102	AT1G01430	exonic	nonsynonymous SNV	Chr1	157407	T	C	TBL25, TRICHOME BIREFRINGENCE-LIKE 25
101/102	AT1G01430	exonic	nonsynonymous SNV	Chr1	157974	C	T	
101/102	AT1G01430	exonic	nonsynonymous SNV	Chr1	158305	A	G	
101/102	AT1G01430	exonic	nonsynonymous SNV	Chr1	158355	G	A	
101/102	AT1G01450	exonic	frameshift deletion	Chr1	164164	CTATATA	CTATA	Protein kinase superfamily protein
101/102	AT1G01450	exonic	nonsynonymous SNV	Chr1	164638	A	C	
101/102	AT1G01450	exonic	nonsynonymous SNV	Chr1	165105	T	G	
101/102	AT1G01450	exonic	stopgain	Chr1	165397	G	A	
101/102	AT1G01453	exonic	nonsynonymous SNV	Chr1	167692	T	G	
101/102	AT1G01460	exonic	nonsynonymous SNV	Chr1	169429	A	T	ATPIPK11, PIPK11
101/102	AT1G01460	exonic	nonsynonymous SNV	Chr1	170271	A	G	
101/102	AT1G01460	exonic	nonsynonymous SNV	Chr1	170284	T	G	
101/102 & 102/103	AT1G01471	exonic	nonsynonymous SNV	Chr1	173351	C	T	
101/102 & 102/103	AT1G01471	exonic	nonsynonymous SNV	Chr1	173359	C	A	
101/102 & 102/103	AT1G01471	exonic	nonsynonymous SNV	Chr1	173387	T	A	
101/102 & 102/103	AT1G01471	exonic	nonsynonymous SNV	Chr1	173392	G	A	
101/102 & 102/103	AT1G01471	exonic	nonsynonymous SNV	Chr1	173417	A	C	
101/102 & 102/103	AT1G01471	exonic	nonsynonymous SNV	Chr1	173428	C	A	
101/102 & 102/103	AT1G01480	exonic	nonsynonymous SNV	Chr1	176276	A	G	1-AMINO-CYCLOPROPANE-1-CARBOXYLATE SYNTHASE 2, ACS2, AT-ACC2
101/102 & 102/103	AT1G01480	exonic	nonsynonymous SNV	Chr1	177089	G	A	
101/102 & 102/103	AT1G01480	exonic	nonsynonymous SNV	Chr1	177090	A	G	
101/102 & 102/103	AT1G01480	exonic	nonsynonymous SNV	Chr1	177112	C	G	
101/102 & 102/103	AT1G01480	exonic	nonsynonymous SNV	Chr1	177117	C	T	
101/102 & 102/103	AT1G01480	exonic	nonsynonymous SNV	Chr1	177121	A	C	
101/102 & 102/103	AT1G01480	exonic	nonsynonymous SNV	Chr1	177611	A	C	

101/102 & 102/103	AT1G01480	exonic	nonsynonymous SNV	Chr1	177741	G	A	
101/102 & 102/103	AT1G01480	exonic	nonsynonymous SNV	Chr1	177909	A	T	
101/102 & 102/103	AT1G01500	exonic	nonsynonymous SNV	Chr1	185783	T	C	
101/102 & 102/103	AT1G01510	exonic	nonsynonymous SNV	Chr1	187761	G	C	AN, ANGUSTIFOLIA, DETORQUEO, DOQ
101/102 & 102/103	AT1G01510	exonic	nonsynonymous SNV	Chr1	187763	T	G	
101/102 & 102/103	AT1G01510	exonic	nonsynonymous SNV	Chr1	187768	A	T	
101/102 & 102/103	AT1G01530	exonic	nonsynonymous SNV	Chr1	193553	C	G	
101/102 & 102/103	AT1G01540	exonic	frameshift insertion	Chr1	197912	A	AT	Protein kinase superfamily protein;(source:Araport11)
101/102 & 102/103	AT1G01540	exonic	nonsynonymous SNV	Chr1	197929	T	C	
101/102 & 102/103	AT1G01540	exonic	nonsynonymous SNV	Chr1	197947	T	G	
101/102 & 102/103	AT1G01540	exonic	nonsynonymous SNV	Chr1	197963	C	T	
101/102 & 102/103	AT1G01540	exonic	nonsynonymous SNV	Chr1	198347	G	C	
101/102 & 102/103	AT1G01540	exonic	nonsynonymous SNV	Chr1	198376	A	T	
101/102 & 102/103	AT1G01550	exonic	nonsynonymous SNV	Chr1	200533	G	C	BPS1, BYPASS 1
101/102 & 102/103	AT1G01550	exonic	nonsynonymous SNV	Chr1	200551	G	C	
101/102 & 102/103	AT1G01550	exonic	stopgain	Chr1	200568	G	T	
101/102 & 102/103	AT1G01570	exonic	nonsynonymous SNV	Chr1	205393	A	G	transferring glycosyl group transferase (DUF604);(source:Araport11)
101/102 & 102/103	AT1G01570	exonic	nonsynonymous SNV	Chr1	205710	A	G	
101/102 & 102/103	AT1G01570	exonic	nonsynonymous SNV	Chr1	207187	T	A	
101/102 & 102/103	AT1G01570	exonic	nonsynonymous SNV	Chr1	207196	G	C	
101/102 & 102/103	AT1G01580	exonic	nonsynonymous SNV	Chr1	210438	T	C	ATFRO2, FERRIC CHELATE REDUCTASE DEFECTIVE 1, FERRIC REDUCTION OXIDASE 2, FRD
101/102 & 102/103	AT1G01580	exonic	nonsynonymous SNV	Chr1	210543	A	C	
101/102 & 102/103	AT1G01580	exonic	nonsynonymous SNV	Chr1	211861	A	C	
101/102 & 102/103	AT1G01590	exonic	nonsynonymous SNV	Chr1	214515	C	G	ATFRO1, FERRIC REDUCTION OXIDASE 1, FRO1
101/102 & 102/103	AT1G01590	exonic	nonsynonymous SNV	Chr1	215411	T	G	
101/102 & 102/103	AT1G01590	exonic	nonsynonymous SNV	Chr1	215414	T	G	
101/102 & 102/103	AT1G01590	exonic	nonsynonymous SNV	Chr1	215421	C	G	
101/102 & 102/103	AT1G01590	exonic	nonsynonymous SNV	Chr1	215427	T	G	
101/102 & 102/103	AT1G01590	exonic	nonsynonymous SNV	Chr1	215432	C	G	
101/102 & 102/103	AT1G01590	exonic	nonsynonymous SNV	Chr1	215435	A	T	
101/102 & 102/103	AT1G01590	exonic	nonsynonymous SNV	Chr1	215444	T	G	
101/102 & 102/103	AT1G01590	exonic	nonsynonymous SNV	Chr1	215677	G	T	
101/102 & 102/103	AT1G01590	exonic	nonsynonymous SNV	Chr1	215760	G	C	

101/102 & 102/103	AT1G01590	exonic	nonsynonymous SNV	Chr1	215890	C	A	
101/102 & 102/103	AT1G01590	exonic	nonsynonymous SNV	Chr1	216643	G	A	
101/102 & 102/103	AT1G01590	exonic	nonsynonymous SNV	Chr1	216875	A	C	
101/102 & 102/103	AT1G01600	exonic	nonsynonymous SNV	Chr1	220860	T	C	"CYTOCHROME P450, FAMILY 86, SUBFAMILY A, POLYPEPTIDE 4", CYP86A4
101/102 & 102/103	AT1G01600	exonic	nonsynonymous SNV	Chr1	220937	C	G	
101/102 & 102/103	AT1G01600	exonic	nonsynonymous SNV	Chr1	220968	T	A	
101/102 & 102/103	AT1G01650	exonic	nonsynonymous SNV	Chr1	233581	A	C	
101/102 & 102/103	AT1G01670	exonic	nonsynonymous SNV	Chr1	244174	C	T	
101/102 & 102/103	AT1G01670	exonic	nonsynonymous SNV	Chr1	244584	C	T	
101/102 & 102/103	AT1G01680	exonic	nonsynonymous SNV	Chr1	247400	G	C	
101/102 & 102/103	AT1G01680	exonic	nonsynonymous SNV	Chr1	247410	A	T	
101/102 & 102/103	AT1G01690	exonic	nonsynonymous SNV	Chr1	249795	G	T	
101/102 & 102/103	AT1G01690	exonic	nonsynonymous SNV	Chr1	249796	A	C	
101/102 & 102/103	AT1G01695	exonic	nonsynonymous SNV	Chr1	252953	G	A	TON1 RECRUITING MOTIF 33, TRM33
101/102 & 102/103	AT1G01695	exonic	nonsynonymous SNV	Chr1	253606	G	A	
101/102 & 102/103	AT1G01695	exonic	nonsynonymous SNV	Chr1	254004	C	G	
101/102 & 102/103	AT1G01700	exonic	nonsynonymous SNV	Chr1	259953	G	C	ATROPGEF2, ROP (RHO OF PLANTS) GUANINE NUCLEOTIDE EXCHANGE FACTOR 2, ROPG
101/102 & 102/103	AT1G01700	exonic	nonsynonymous SNV	Chr1	260010	C	A	
101/102 & 102/103	AT1G01700	exonic	nonsynonymous SNV	Chr1	260158	T	C	
101/102 & 102/103	AT1G01710	exonic	nonsynonymous SNV	Chr1	265611	G	C	
101/102 & 102/103	AT1G01720	exonic	nonsynonymous SNV	Chr1	268852	T	G	
101/102 & 102/103	AT1G01730	exonic	nonsynonymous SNV	Chr1	271050	G	C	
101/102 & 102/103	AT1G01730	exonic	nonsynonymous SNV	Chr1	271416	T	C	
101/102 & 102/103	AT1G01740	exonic	nonsynonymous SNV	Chr1	272826	G	A	
101/102 & 102/103	AT1G01770	exonic	nonsynonymous SNV	Chr1	281313	G	A	
101/102 & 102/103	AT1G01770	exonic	nonsynonymous SNV	Chr1	281933	G	C	
101/102 & 102/103	AT1G01790	exonic	nonsynonymous SNV	Chr1	284989	A	G	ATKEA1, K+ EFFLUX ANTIPORTER 1, KEA1
101/102 & 102/103	AT1G01790	exonic	nonsynonymous SNV	Chr1	285111	G	A	
101/102 & 102/103	AT1G01790	exonic	nonsynonymous SNV	Chr1	285729	A	G	
101/102 & 102/103	AT1G01790	exonic	nonsynonymous SNV	Chr1	286029	A	C	
101/102 & 102/103	AT1G01800	exonic	nonsynonymous SNV	Chr1	294545	G	C	
101/102 & 102/103	AT1G01800	exonic	nonsynonymous SNV	Chr1	294704	C	G	
101/102 & 102/103	AT1G01820	exonic	nonsynonymous SNV	Chr1	296955	T	A	PEROXIN 11C, PEX11C

101/102 & 102/103	AT1G01820	exonic	nonsynonymous SNV	Chr1	296959	T	C	
101/102 & 102/103	AT1G01820	exonic	nonsynonymous SNV	Chr1	296965	T	C	
102/103 & 103/104	AT1G01830	exonic	nonsynonymous SNV	Chr1	299439	T	C	ARM repeat superfamily protein
102/103 & 103/104	AT1G01830	exonic	nonsynonymous SNV	Chr1	299440	T	C	
102/103 & 103/104	AT1G01830	exonic	nonsynonymous SNV	Chr1	300023	C	A	
102/103 & 103/104	AT1G01830	exonic	nonsynonymous SNV	Chr1	300024	C	T	
102/103 & 103/104	AT1G01830	exonic	nonsynonymous SNV	Chr1	300277	C	A	
102/103 & 103/104	AT1G01880	exonic	nonsynonymous SNV	Chr1	306651	A	C	ATGEN1, GEN1, ORTHOLOG OF HSGEN1
102/103 & 103/104	AT1G01880	exonic	nonsynonymous SNV	Chr1	306653	T	C	
102/103 & 103/104	AT1G01880	exonic	nonsynonymous SNV	Chr1	307266	C	T	
102/103 & 103/104	AT1G01900	exonic	nonsynonymous SNV	Chr1	310748	G	T	
102/103 & 103/104	AT1G01930	exonic	nonsynonymous SNV	Chr1	321016	G	A	
102/103 & 103/104	AT1G01930	exonic	nonsynonymous SNV	Chr1	321370	C	A	
102/103 & 103/104	AT1G01950	exonic	nonsynonymous SNV	Chr1	326964	T	G	ARABIDOPSIS THALIANA KINESIN UNGROUPED CLADE, GENE B, ARK2, ARMADILLO REPE
102/103 & 103/104	AT1G01950	exonic	nonsynonymous SNV	Chr1	326965	T	A	
102/103 & 103/104	AT1G01950	exonic	nonsynonymous SNV	Chr1	328445	A	T	
102/103 & 103/104	AT1G01960	exonic	nonsynonymous SNV	Chr1	331269	A	G	BIG3, EDA10, EMBRYO SAC DEVELOPMENT ARREST 10
102/103 & 103/104	AT1G01960	exonic	nonsynonymous SNV	Chr1	331736	A	C	
102/103 & 103/104	AT1G01960	exonic	nonsynonymous SNV	Chr1	332347	A	T	
102/103 & 103/104	AT1G01970	exonic	nonsynonymous SNV	Chr1	338744	T	G	
102/103 & 103/104	AT1G01980	exonic	nonsynonymous SNV	Chr1	340810	C	A	ATBBE1
102/103 & 103/104	AT1G01980	exonic	nonsynonymous SNV	Chr1	341545	G	A	
102/103 & 103/104	AT1G01980	exonic	nonsynonymous SNV	Chr1	341924	T	C	
102/103 & 103/104	AT1G01980	exonic	nonsynonymous SNV	Chr1	341969	C	A	
102/103 & 103/104	AT1G01980	exonic	nonsynonymous SNV	Chr1	341982	C	A	
102/103 & 103/104	AT1G01980	exonic	nonsynonymous SNV	Chr1	341990	A	C	
102/103 & 103/104	AT1G01990	exonic	nonsynonymous SNV	Chr1	344075	G	A	
102/103 & 103/104	AT1G02010	exonic	nonsynonymous SNV	Chr1	349310	C	A	
102/103 & 103/104	AT1G02020	exonic	frameshift insertion	Chr1	354813	T	TACTT	nitroreductase family protein
102/103 & 103/104	AT1G02020	exonic	nonsynonymous SNV	Chr1	353491	A	T	
102/103 & 103/104	AT1G02020	exonic	nonsynonymous SNV	Chr1	354413	G	C	
102/103 & 103/104	AT1G02020	exonic	nonsynonymous SNV	Chr1	354473	C	T	
102/103 & 103/104	AT1G02020	exonic	nonsynonymous SNV	Chr1	354700	G	A	

102/103 & 103/104	AT1G02020	exonic	nonsynonymous SNV	Chr1	354701	A	C	
102/103 & 103/104	AT1G02020	exonic	nonsynonymous SNV	Chr1	354727	A	G	
102/103 & 103/104	AT1G02020	exonic	nonsynonymous SNV	Chr1	354746	G	A	
102/103 & 103/104	AT1G02030	exonic	nonsynonymous SNV	Chr1	356127	C	T	
102/103 & 103/104	AT1G02040	exonic	nonsynonymous SNV	Chr1	358397	T	G	C2H2-type zinc finger family protein
102/103 & 103/104	AT1G02040	exonic	nonsynonymous SNV	Chr1	358570	G	T	
102/103 & 103/104	AT1G02040	exonic	nonsynonymous SNV	Chr1	358639	G	C	
102/103 & 103/104	AT1G02050	exonic	nonsynonymous SNV	Chr1	359566	C	G	
102/103 & 103/104	AT1G02060	exonic	frameshift deletion	Chr1	361050	GT	G	Tetratricopeptide repeat (TPR)-like superfamily protein
102/103 & 103/104	AT1G02060	exonic	nonsynonymous SNV	Chr1	361859	G	C	
102/103 & 103/104	AT1G02060	exonic	nonsynonymous SNV	Chr1	362150	C	A	
102/103 & 103/104	AT1G02065	exonic	nonsynonymous SNV	Chr1	365823	C	T	
102/103 & 103/104	AT1G02065	exonic	nonsynonymous SNV	Chr1	366788	A	C	
102/103 & 103/104	AT1G02080	exonic	nonsynonymous SNV	Chr1	373680	G	T	transcription regulator
102/103 & 103/104	AT1G02080	exonic	nonsynonymous SNV	Chr1	375346	G	A	
102/103 & 103/104	AT1G02080	exonic	nonsynonymous SNV	Chr1	381773	A	T	
102/103 & 103/104	AT1G02080	exonic	nonsynonymous SNV	Chr1	384704	A	T	
102/103 & 103/104	AT1G02110	exonic	nonsynonymous SNV	Chr1	393497	C	T	bZIP domain class transcription factor (DUF630 and DUF632)
102/103 & 103/104	AT1G02110	exonic	nonsynonymous SNV	Chr1	395265	T	G	
102/103 & 103/104	AT1G02110	exonic	nonsynonymous SNV	Chr1	395277	T	G	
102/103 & 103/104	AT1G02120	exonic	nonsynonymous SNV	Chr1	395878	A	G	
102/103 & 103/104	AT1G02120	exonic	nonsynonymous SNV	Chr1	399654	A	T	
102/103 & 103/104	AT1G02140	exonic	nonsynonymous SNV	Chr1	404233	T	G	
102/103 & 103/104	AT1G02145	exonic	nonsynonymous SNV	Chr1	405652	A	G	
102/103 & 103/104	AT1G02150	exonic	nonsynonymous SNV	Chr1	408849	T	G	
102/103 & 103/104	AT1G02180	exonic	nonsynonymous SNV	Chr1	413624	A	G	ferredoxin-like protein
102/103 & 103/104	AT1G02180	exonic	nonsynonymous SNV	Chr1	414477	A	G	
102/103 & 103/104	AT1G02180	exonic	nonsynonymous SNV	Chr1	414481	C	T	
102/103 & 103/104	AT1G02180	exonic	nonsynonymous SNV	Chr1	414484	A	T	
102/103 & 103/104	AT1G02180	exonic	nonsynonymous SNV	Chr1	414487	T	A	
102/103 & 103/104	AT1G02190	exonic	nonsynonymous SNV	Chr1	416141	C	G	Fatty acid hydroxylase superfamily
102/103 & 103/104	AT1G02190	exonic	nonsynonymous SNV	Chr1	417318	C	G	
102/103 & 103/104	AT1G02190	exonic	nonsynonymous SNV	Chr1	417642	T	G	

102/103 & 103/104	AT1G02190	exonic	nonsynonymous SNV	Chr1	417789	C	A	
102/103 & 103/104	AT1G02205	exonic	nonsynonymous SNV	Chr1	420807	A	C	CER1, CER22, ECERIFERUM 1, ECERIFERUM 22
102/103 & 103/104	AT1G02205	exonic	nonsynonymous SNV	Chr1	420866	G	A	
102/103 & 103/104	AT1G02205	exonic	nonsynonymous SNV	Chr1	421745	T	G	
102/103 & 103/104	AT1G02205	exonic	nonsynonymous SNV	Chr1	422054	T	C	
102/103 & 103/104	AT1G02205	exonic	nonsynonymous SNV	Chr1	422095	C	A	
111/112	AT1G04130	exonic	nonsynonymous SNV	Chr1	1073941	C	G	
111/112	AT1G04140	exonic	nonsynonymous SNV	Chr1	1077510	C	T	
111/112	AT1G04140	exonic	nonsynonymous SNV	Chr1	1080239	C	T	
111/112	AT1G04160	exonic	nonsynonymous SNV	Chr1	1087192	G	T	
111/112	AT1G04160	exonic	nonsynonymous SNV	Chr1	1088730	C	A	
111/112	AT1G04160	exonic	nonsynonymous SNV	Chr1	1089125	A	G	
111/112	AT1G04160	exonic	nonsynonymous SNV	Chr1	1092049	T	C	
111/112	AT1G04160	exonic	nonsynonymous SNV	Chr1	1092888	C	T	
111/112	AT1G04160	exonic	nonsynonymous SNV	Chr1	1093364	A	G	
111/112	AT1G04160	exonic	nonsynonymous SNV	Chr1	1093415	T	A	
111/112	AT1G04160	exonic	nonsynonymous SNV	Chr1	1093442	A	G	
111/112	AT1G04160	exonic	nonsynonymous SNV	Chr1	1093696	T	C	
111/112	AT1G04160	exonic	nonsynonymous SNV	Chr1	1094402	G	A	
111/112	AT1G04171	exonic	nonsynonymous SNV	Chr1	1100483	C	G	
111/112	AT1G04171	exonic	stoploss	Chr1	1100445	T	C	
111/112	AT1G04180	exonic	nonsynonymous SNV	Chr1	1105035	A	C	
111/112	AT1G04180	exonic	nonsynonymous SNV	Chr1	1105284	G	A	
111/112	AT1G04180	exonic	nonsynonymous SNV	Chr1	1105473	G	C	
111/112	AT1G04180	exonic	nonsynonymous SNV	Chr1	1105494	T	A	
111/112	AT1G04190	exonic	nonsynonymous SNV	Chr1	1108545	C	A	
111/112	AT1G04200	exonic	nonsynonymous SNV	Chr1	1112557	G	T	
111/112	AT1G04210	exonic	nonsynonymous SNV	Chr1	1114803	C	G	
111/112	AT1G04210	exonic	nonsynonymous SNV	Chr1	1115603	G	A	
111/112	AT1G04210	exonic	nonsynonymous SNV	Chr1	1116274	G	T	
111/112	AT1G04210	exonic	nonsynonymous SNV	Chr1	1116487	G	T	

111/112	AT1G04210	exonic	nonsynonymous SNV	Chr1	1116814	G	A	
111/112	AT1G04220	exonic	nonsynonymous SNV	Chr1	1121987	A	G	
111/112	AT1G04230	exonic	nonsynonymous SNV	Chr1	1126079	T	G	
111/112	AT1G04280	exonic	nonsynonymous SNV	Chr1	1143832	A	C	
111/112	AT1G04280	exonic	nonsynonymous SNV	Chr1	1144374	C	G	
111/112	AT1G04280	exonic	nonsynonymous SNV	Chr1	1144388	T	G	
111/112	AT1G04280	exonic	nonsynonymous SNV	Chr1	1144395	T	A	
111/112	AT1G04280	exonic	nonsynonymous SNV	Chr1	1144396	T	G	
111/112	AT1G04300	exonic	nonsynonymous SNV	Chr1	1149308	T	G	
111/112	AT1G04300	exonic	nonsynonymous SNV	Chr1	1149464	C	A	
111/112	AT1G04300	exonic	nonsynonymous SNV	Chr1	1149753	C	G	
111/112	AT1G04310	exonic	nonsynonymous SNV	Chr1	1155448	T	C	
111/112	AT1G04310	exonic	nonsynonymous SNV	Chr1	1155536	G	C	
111/112	AT1G04310	exonic	nonsynonymous SNV	Chr1	1155601	G	T	
111/112	AT1G04310	exonic	nonsynonymous SNV	Chr1	1155940	A	G	
111/112	AT1G04330	exonic	nonsynonymous SNV	Chr1	1161615	G	A	
111/112	AT1G04350	exonic	nonsynonymous SNV	Chr1	1165444	T	C	
111/112	AT1G04360	exonic	nonsynonymous SNV	Chr1	1167844	C	T	
111/112	AT1G04360	exonic	nonsynonymous SNV	Chr1	1168048	T	C	
111/112	AT1G04370	exonic	nonsynonymous SNV	Chr1	1175414	T	A	
111/112	AT1G04380	exonic	frameshift insertion	Chr1	1177257	A	AG	
111/112	AT1G04380	exonic	nonsynonymous SNV	Chr1	1177976	C	A	
111/112	AT1G04380	exonic	nonsynonymous SNV	Chr1	1178065	T	A	
111/112	AT1G04380	exonic	nonsynonymous SNV	Chr1	1178252	C	T	
111/112	AT1G04380	exonic	nonsynonymous SNV	Chr1	1178349	C	G	
111/112	AT1G04390	exonic	nonsynonymous SNV	Chr1	1182175	G	A	
111/112	AT1G04390	exonic	nonsynonymous SNV	Chr1	1183153	T	A	
111/112	AT1G04400	exonic	nonsynonymous SNV	Chr1	1186131	G	A	
111/112	AT1G04400	exonic	nonsynonymous SNV	Chr1	1186604	C	T	CRY2
111/112	AT1G04440	exonic	nonsynonymous SNV	Chr1	1205599	G	A	
111/112	AT1G04445	exonic	nonsynonymous SNV	Chr1	1207461	T	C	
111/112	AT1G04445	exonic	nonsynonymous SNV	Chr1	1207631	T	G	
111/112	AT1G04470	exonic	nonsynonymous SNV	Chr1	1211516	C	G	

111/112	AT1G04470	exonic	nonsynonymous SNV	Chr1	1211716	G	T
111/112	AT1G04470	exonic	nonsynonymous SNV	Chr1	1212827	A	C
111/112	AT1G04470	exonic	nonsynonymous SNV	Chr1	1213602	G	T
111/112	AT1G04500	exonic	nonsynonymous SNV	Chr1	1221994	G	T
111/112	AT1G04500	exonic	nonsynonymous SNV	Chr1	1222002	G	T
111/112	AT1G04500	exonic	nonsynonymous SNV	Chr1	1222664	G	A
111/112	AT1G04500	exonic	nonsynonymous SNV	Chr1	1222848	G	A
111/112	AT1G04500	exonic	nonsynonymous SNV	Chr1	1222849	T	A
111/112	AT1G04501	exonic	frameshift insertion	Chr1	1224678	T	TG
111/112	AT1G04510	exonic	nonsynonymous SNV	Chr1	1230313	G	A
111/112	AT1G04510	exonic	nonsynonymous SNV	Chr1	1230476	A	T
111/112	AT1G04530	exonic	nonsynonymous SNV	Chr1	1234740	C	T
111/112	AT1G04540	exonic	nonsynonymous SNV	Chr1	1238041	G	T
111/112	AT1G04540	exonic	nonsynonymous SNV	Chr1	1238146	C	A
111/112	AT1G04540	exonic	nonsynonymous SNV	Chr1	1238148	A	C
111/112	AT1G04540	exonic	nonsynonymous SNV	Chr1	1238210	A	T
111/112	AT1G04550	exonic	nonsynonymous SNV	Chr1	1241208	C	G
111/112	AT1G04580	exonic	nonsynonymous SNV	Chr1	1252247	T	C
111/112	AT1G04580	exonic	nonsynonymous SNV	Chr1	1252557	C	T
111/112	AT1G04580	exonic	nonsynonymous SNV	Chr1	1253047	C	T
111/112	AT1G04580	exonic	nonsynonymous SNV	Chr1	1253643	C	T
111/112	AT1G04580	exonic	nonsynonymous SNV	Chr1	1253963	G	A
111/112	AT1G04580	exonic	nonsynonymous SNV	Chr1	1253964	A	T
111/112	AT1G04580	exonic	nonsynonymous SNV	Chr1	1253977	A	T
111/112	AT1G04580	exonic	nonsynonymous SNV	Chr1	1253989	G	C
111/112	AT1G04580	exonic	nonsynonymous SNV	Chr1	1256134	T	C
111/112	AT1G04580	exonic	nonsynonymous SNV	Chr1	1256399	T	C
111/112	AT1G04580	exonic	nonsynonymous SNV	Chr1	1256409	C	A
111/112	AT1G04580	exonic	nonsynonymous SNV	Chr1	1257491	A	C
111/112	AT1G04590	exonic	nonsynonymous SNV	Chr1	1258774	C	T
111/112	AT1G04590	exonic	nonsynonymous SNV	Chr1	1258830	C	A
111/112	AT1G04590	exonic	nonsynonymous SNV	Chr1	1258841	G	C
111/112	AT1G04590	exonic	nonsynonymous SNV	Chr1	1258843	T	A

111/112	AT1G04590	exonic	nonsynonymous SNV	Chr1	1259468	C	T	
111/112	AT1G04590	exonic	nonsynonymous SNV	Chr1	1260778	C	A	
111/112	AT1G04590	exonic	nonsynonymous SNV	Chr1	1260784	C	T	
111/112	AT1G04590	exonic	nonsynonymous SNV	Chr1	1260793	C	T	
111/112	AT1G04600	exonic	nonsynonymous SNV	Chr1	1262584	C	G	
111/112	AT1G04600	exonic	nonsynonymous SNV	Chr1	1264578	G	A	
111/112	AT1G04600	exonic	nonsynonymous SNV	Chr1	1265645	G	T	
111/112	AT1G04600	exonic	nonsynonymous SNV	Chr1	1265875	G	A	
111/112	AT1G04600	exonic	nonsynonymous SNV	Chr1	1266414	C	T	
111/112	AT1G04600	exonic	nonsynonymous SNV	Chr1	1268742	A	C	
111/112	AT1G04600	exonic	nonsynonymous SNV	Chr1	1269300	G	A	
111/112	AT1G04600	exonic	nonsynonymous SNV	Chr1	1270934	T	C	
111/112	AT1G04600	exonic	nonsynonymous SNV	Chr1	1270935	C	T	
111/112	AT1G04600	exonic	nonsynonymous SNV	Chr1	1270940	A	G	
111/112	AT1G04600	exonic	nonsynonymous SNV	Chr1	1270941	A	C	
111/112	AT1G04600	exonic	nonsynonymous SNV	Chr1	1270942	G	T	
111/112	AT1G04600	exonic	nonsynonymous SNV	Chr1	1270943	A	G	
111/112	AT1G04600	exonic	nonsynonymous SNV	Chr1	1270953	A	G	
111/112	AT1G04600	exonic	nonsynonymous SNV	Chr1	1270957	G	T	
111/112	AT1G04600	exonic	nonsynonymous SNV	Chr1	1271564	G	A	
111/112	AT1G04600	exonic	nonsynonymous SNV	Chr1	1271577	C	A	
111/112	AT1G04610	exonic	nonsynonymous SNV	Chr1	1279957	T	G	
111/112	AT1G04625	exonic	nonsynonymous SNV	Chr1	1288684	T	C	
111/112	AT1G04630	exonic	nonsynonymous SNV	Chr1	1290815	C	A	
115/116	AT1G05180	exonic	nonsynonymous SNV	Chr1	1498549	A	G	
115/116	AT1G05180	exonic	nonsynonymous SNV	Chr1	1500228	C	T	
115/116	AT1G05200	exonic	nonsynonymous SNV	Chr1	1505708	G	C	ATGLR3.4, GLR3.4, GLUR3, GLUTAMATE RECEPTOR 3.4
115/116	AT1G05200	exonic	nonsynonymous SNV	Chr1	1505757	T	A	
115/116	AT1G05200	exonic	nonsynonymous SNV	Chr1	1505796	G	C	
115/116	AT1G05200	exonic	nonsynonymous SNV	Chr1	1505805	G	C	
115/116	AT1G05200	exonic	nonsynonymous SNV	Chr1	1505919	G	C	

115/116	AT1G05220	exonic	nonsynonymous SNV	Chr1	1512531	G	A	
115/116	AT1G05220	exonic	nonsynonymous SNV	Chr1	1512630	G	C	
115/116	AT1G05230	exonic	nonsynonymous SNV	Chr1	1515063	T	C	
115/116	AT1G05260	exonic	nonsynonymous SNV	Chr1	1529880	T	G	
115/116	AT1G05270	exonic	nonsynonymous SNV	Chr1	1532755	A	C	TraB family protein
115/116	AT1G05270	exonic	nonsynonymous SNV	Chr1	1534106	T	C	
115/116	AT1G05270	exonic	nonsynonymous SNV	Chr1	1534298	A	T	
115/116	AT1G05280	exonic	nonsynonymous SNV	Chr1	1535468	A	C	ERV-F (C)1 provirus ancestral Env polyprotein, putative (DUF604)
115/116	AT1G05280	exonic	nonsynonymous SNV	Chr1	1536067	G	C	
115/116	AT1G05280	exonic	nonsynonymous SNV	Chr1	1536136	C	A	
115/116	AT1G05280	exonic	nonsynonymous SNV	Chr1	1537170	A	T	
115/116	AT1G05290	exonic	nonsynonymous SNV	Chr1	1539258	G	C	CCT motif family protein
115/116	AT1G05290	exonic	nonsynonymous SNV	Chr1	1539465	G	A	
115/116	AT1G05290	exonic	nonsynonymous SNV	Chr1	1539666	G	A	
115/116	AT1G05290	exonic	nonsynonymous SNV	Chr1	1539810	C	T	
115/116	AT1G05290	exonic	nonsynonymous SNV	Chr1	1539823	C	G	
115/116	AT1G05290	exonic	nonsynonymous SNV	Chr1	1540311	C	A	
115/116	AT1G05290	exonic	nonsynonymous SNV	Chr1	1540545	A	T	
115/116	AT1G05300	exonic	nonsynonymous SNV	Chr1	1545548	T	C	
115/116	AT1G05300	exonic	nonsynonymous SNV	Chr1	1547262	C	A	
115/116	AT1G05310	exonic	nonsynonymous SNV	Chr1	1550655	A	T	Pectin lyase-like superfamily protein
115/116	AT1G05310	exonic	nonsynonymous SNV	Chr1	1552172	G	A	
115/116	AT1G05310	exonic	nonsynonymous SNV	Chr1	1552179	C	T	
115/116	AT1G05320	exonic	nonsynonymous SNV	Chr1	1555291	C	T	
115/116	AT1G05320	exonic	nonsynonymous SNV	Chr1	1556199	C	G	
115/116	AT1G05320	exonic	nonsynonymous SNV	Chr1	1557299	A	G	
115/116	AT1G05320	exonic	nonsynonymous SNV	Chr1	1557641	G	C	
115/116	AT1G05350	exonic	nonsynonymous SNV	Chr1	1563930	T	G	
115/116	AT1G05360	exonic	nonsynonymous SNV	Chr1	1565881	T	C	KILLING ME SLOWLY 2, KMS2
115/116	AT1G05360	exonic	nonsynonymous SNV	Chr1	1565916	A	G	
115/116	AT1G05360	exonic	nonsynonymous SNV	Chr1	1566911	G	A	
115/116	AT1G05360	exonic	nonsynonymous SNV	Chr1	1567259	C	G	
115/116	AT1G05370	exonic	nonsynonymous SNV	Chr1	1569615	T	C	myosin heavy chain, embryonic smooth protein

115/116	AT1G05370	exonic	nonsynonymous SNV	Chr1	1569666	T	G	
115/116	AT1G05370	exonic	nonsynonymous SNV	Chr1	1569677	T	G	
115/116	AT1G05370	exonic	nonsynonymous SNV	Chr1	1569687	T	G	
115/116	AT1G05380	exonic	nonsynonymous SNV	Chr1	1578543	C	G	Acyl-CoA N-acyltransferase with RING/FYVE/PHD-type zinc finger protein
115/116	AT1G05380	exonic	nonsynonymous SNV	Chr1	1579543	T	C	
115/116	AT1G05380	exonic	nonsynonymous SNV	Chr1	1580237	G	A	
115/116	AT1G05380	exonic	nonsynonymous SNV	Chr1	1581606	G	A	
115/116	AT1G05410	exonic	nonsynonymous SNV	Chr1	1585517	T	C	CDPK adapter, putative (DUF1423)
115/116	AT1G05410	exonic	nonsynonymous SNV	Chr1	1585637	T	G	
115/116	AT1G05410	exonic	nonsynonymous SNV	Chr1	1586313	A	C	
115/116	AT1G05410	exonic	nonsynonymous SNV	Chr1	1586415	T	C	
115/116	AT1G05410	exonic	nonsynonymous SNV	Chr1	1586980	G	T	
115/116	AT1G05420	exonic	nonsynonymous SNV	Chr1	1590148	G	A	ARABIDOPSIS THALIANA OVATE FAMILY PROTEIN 12, ATOFP12, OFP12, OVATE FAMILY P
115/116	AT1G05420	exonic	nonsynonymous SNV	Chr1	1590175	C	A	
115/116	AT1G05420	exonic	nonsynonymous SNV	Chr1	1590233	A	C	
115/116	AT1G05420	exonic	nonsynonymous SNV	Chr1	1590409	T	C	
115/116	AT1G05420	exonic	nonsynonymous SNV	Chr1	1590589	A	C	
115/116	AT1G05430	exonic	nonsynonymous SNV	Chr1	1594587	C	T	hypothetical protein
115/116	AT1G05430	exonic	nonsynonymous SNV	Chr1	1594594	T	G	
115/116	AT1G05430	exonic	nonsynonymous SNV	Chr1	1594628	T	A	
115/116	AT1G05430	exonic	nonsynonymous SNV	Chr1	1595032	A	T	
115/116	AT1G05440	exonic	nonsynonymous SNV	Chr1	1596493	C	T	
115/116	AT1G05440	exonic	nonsynonymous SNV	Chr1	1597764	G	A	
115/116	AT1G05460	exonic	nonsynonymous SNV	Chr1	1602829	G	A	SDE3, SILENCING DEFECTIVE
115/116	AT1G05460	exonic	nonsynonymous SNV	Chr1	1603341	T	C	
115/116	AT1G05460	exonic	nonsynonymous SNV	Chr1	1603459	A	G	
115/116	AT1G05460	exonic	nonsynonymous SNV	Chr1	1603471	T	A	
115/116	AT1G05470	exonic	nonsynonymous SNV	Chr1	1610192	T	C	
115/116	AT1G05490	exonic	nonsynonymous SNV	Chr1	1620624	T	C	
115/116	AT1G05490	exonic	nonsynonymous SNV	Chr1	1621838	T	A	
115/116	AT1G05500	exonic	nonsynonymous SNV	Chr1	1625751	A	C	
115/116	AT1G05520	exonic	nonsynonymous SNV	Chr1	1631841	A	C	
115/116	AT1G05550	exonic	nonsynonymous SNV	Chr1	1641504	C	T	

115/116	AT1G05550	exonic	nonsynonymous SNV	Chr1	1642614	G	A	
115/116	AT1G05570	exonic	nonsynonymous SNV	Chr1	1650886	A	C	
115/116	AT1G05570	exonic	nonsynonymous SNV	Chr1	1655859	A	T	
115/116	AT1G05575	exonic	nonsynonymous SNV	Chr1	1662071	T	C	
115/116	AT1G05590	exonic	nonsynonymous SNV	Chr1	1670227	A	C	ATHEX3, BETA-HEXOSAMINIDASE 2, BETA-HEXOSAMINIDASE 3, HEXO2
115/116	AT1G05590	exonic	nonsynonymous SNV	Chr1	1670564	G	C	
115/116	AT1G05590	exonic	nonsynonymous SNV	Chr1	1671273	T	G	
115/116	AT1G05610	exonic	nonsynonymous SNV	Chr1	1674739	T	A	
115/116	AT1G05610	exonic	nonsynonymous SNV	Chr1	1675823	T	C	
115/116	AT1G05615	exonic	nonsynonymous SNV	Chr1	1677548	T	A	B3 domain protein
115/116	AT1G05615	exonic	nonsynonymous SNV	Chr1	1677914	A	C	
115/116	AT1G05615	exonic	nonsynonymous SNV	Chr1	1678264	C	T	
115/116	AT1G05620	exonic	nonsynonymous SNV	Chr1	1680254	G	A	
115/116	AT1G05630	exonic	nonsynonymous SNV	Chr1	1682798	A	C	5PTASE13, ARABIDOPSIS THALIANA INOSITOL-POLYPHOSPHATE 5-PHOSPHATASE 13, AT
115/116	AT1G05630	exonic	nonsynonymous SNV	Chr1	1683963	T	G	
115/116	AT1G05630	exonic	nonsynonymous SNV	Chr1	1686860	T	A	
115/116	AT1G05640	exonic	nonsynonymous SNV	Chr1	1688098	C	T	
115/116	AT1G05650	exonic	nonsynonymous SNV	Chr1	1690472	G	C	
115/116	AT1G05650	exonic	nonsynonymous SNV	Chr1	1691013	A	T	
115/116	AT1G05660	exonic	nonsynonymous SNV	Chr1	1695029	G	A	
115/116	AT1G05660	exonic	nonsynonymous SNV	Chr1	1695902	C	T	
115/116	AT1G05670	exonic	nonsynonymous SNV	Chr1	1698662	T	C	Pentatricopeptide repeat (PPR-like) superfamily protein
115/116	AT1G05670	exonic	nonsynonymous SNV	Chr1	1699068	C	T	
115/116	AT1G05670	exonic	nonsynonymous SNV	Chr1	1699680	A	G	
115/116	AT1G05675	exonic	nonsynonymous SNV	Chr1	1701334	G	C	UDP-Glycosyltransferase superfamily protein
115/116	AT1G05675	exonic	nonsynonymous SNV	Chr1	1701577	G	C	
115/116	AT1G05675	exonic	nonsynonymous SNV	Chr1	1702443	C	G	
115/116	AT1G05680	exonic	nonsynonymous SNV	Chr1	1703332	T	C	UGT74E2, URIDINE DIPHOSPHATE GLYCOSYLTRANSFERASE 74E2
115/116	AT1G05680	exonic	nonsynonymous SNV	Chr1	1703338	C	G	
115/116	AT1G05680	exonic	nonsynonymous SNV	Chr1	1703345	T	C	
125/126	AT1G08060	exonic	nonsynonymous SNV	Chr1	2509854	G	A	

125/126	AT1G08065	exonic	nonsynonymous SNV	Chr1	2512212	A	T	ACA5, ALPHA CARBONIC ANHYDRASE 5, ATACA5
125/126	AT1G08065	exonic	nonsynonymous SNV	Chr1	2512258	C	T	
125/126	AT1G08065	exonic	nonsynonymous SNV	Chr1	2512977	C	A	
125/126	AT1G08065	exonic	nonsynonymous SNV	Chr1	2512993	C	T	
125/126	AT1G08070	exonic	nonsynonymous SNV	Chr1	2515041	C	T	EMB3102, EMBRYO DEFECTIVE 3102, ORGANELLE TRANSCRIPT PROCESSING 82, OTP82
125/126	AT1G08070	exonic	nonsynonymous SNV	Chr1	2515968	G	A	
125/126	AT1G08070	exonic	nonsynonymous SNV	Chr1	2516044	C	T	
125/126	AT1G08070	exonic	nonsynonymous SNV	Chr1	2516326	T	C	
125/126	AT1G08070	exonic	nonsynonymous SNV	Chr1	2516397	A	C	
125/126	AT1G08080	exonic	nonsynonymous SNV	Chr1	2518362	C	T	A. THALIANA ALPHA CARBONIC ANHYDRASE 7, ACA7, ALPHA CARBONIC ANHYDRASE 7, [illegible]
125/126	AT1G08080	exonic	nonsynonymous SNV	Chr1	2518462	A	T	
125/126	AT1G08080	exonic	nonsynonymous SNV	Chr1	2518485	A	G	
125/126	AT1G08080	exonic	nonsynonymous SNV	Chr1	2518514	G	C	
125/126	AT1G08080	exonic	nonsynonymous SNV	Chr1	2518523	A	T	
125/126	AT1G08100	exonic	nonsynonymous SNV	Chr1	2527436	G	C	
125/126	AT1G08110	exonic	nonsynonymous SNV	Chr1	2535690	T	C	ATGLYI2, GLYOXALASE12
125/126	AT1G08110	exonic	nonsynonymous SNV	Chr1	2535699	T	C	
125/126	AT1G08110	exonic	nonsynonymous SNV	Chr1	2535714	G	T	
125/126	AT1G08110	exonic	nonsynonymous SNV	Chr1	2535715	C	T	
125/126	AT1G08125	exonic	nonsynonymous SNV	Chr1	2540106	C	G	S-adenosyl-L-methionine-dependent methyltransferases superfamily protein
125/126	AT1G08125	exonic	nonsynonymous SNV	Chr1	2542131	A	G	
125/126	AT1G08125	exonic	nonsynonymous SNV	Chr1	2542161	A	C	
125/126	AT1G08130	exonic	nonsynonymous SNV	Chr1	2543632	G	A	ATLIG1, DNA LIGASE 1, LIG1
125/126	AT1G08130	exonic	nonsynonymous SNV	Chr1	2546066	C	A	
125/126	AT1G08130	exonic	nonsynonymous SNV	Chr1	2546069	C	G	
125/126	AT1G08130	exonic	nonsynonymous SNV	Chr1	2547316	G	C	
125/126	AT1G08130	exonic	nonsynonymous SNV	Chr1	2547317	A	T	
125/126	AT1G08130	exonic	nonsynonymous SNV	Chr1	2547318	C	A	
125/126	AT1G08130	exonic	nonsynonymous SNV	Chr1	2547320	T	C	
125/126	AT1G08130	exonic	nonsynonymous SNV	Chr1	2547459	A	T	
125/126	AT1G08130	exonic	nonsynonymous SNV	Chr1	2547485	G	C	
125/126	AT1G08130	exonic	nonsynonymous SNV	Chr1	2547487	A	G	
125/126	AT1G08130	exonic	nonsynonymous SNV	Chr1	2547506	G	A	

125/126	AT1G08130	exonic	nonsynonymous SNV	Chr1	2547518	A	G	
125/126	AT1G08130	exonic	nonsynonymous SNV	Chr1	2547688	G	A	
125/126	AT1G08130	exonic	nonsynonymous SNV	Chr1	2547721	A	C	
125/126	AT1G08130	exonic	nonsynonymous SNV	Chr1	2547728	T	G	
125/126	AT1G08130	exonic	nonsynonymous SNV	Chr1	2547743	A	C	
125/126	AT1G08130	exonic	nonsynonymous SNV	Chr1	2547759	T	G	
125/126	AT1G08135	exonic	nonsynonymous SNV	Chr1	2548992	A	C	ATCHX6B, CATION/H+ EXCHANGER 6B, CHX6B
125/126	AT1G08135	exonic	nonsynonymous SNV	Chr1	2549136	A	G	
125/126	AT1G08135	exonic	nonsynonymous SNV	Chr1	2549711	G	T	
125/126	AT1G08135	exonic	nonsynonymous SNV	Chr1	2549914	A	T	
125/126	AT1G08135	exonic	nonsynonymous SNV	Chr1	2550447	G	T	
125/126	AT1G08135	exonic	nonsynonymous SNV	Chr1	2550594	T	G	
125/126	AT1G08135	exonic	nonsynonymous SNV	Chr1	2551032	A	C	
125/126	AT1G08135	exonic	nonsynonymous SNV	Chr1	2551048	C	A	
125/126	AT1G08135	exonic	nonsynonymous SNV	Chr1	2551440	A	G	
125/126	AT1G08140	exonic	nonsynonymous SNV	Chr1	2552226	G	C	ATCHX6A, CATION/H+ EXCHANGER 6A, CHX6A
125/126	AT1G08140	exonic	nonsynonymous SNV	Chr1	2552246	T	C	
125/126	AT1G08140	exonic	nonsynonymous SNV	Chr1	2553324	T	G	
125/126	AT1G08140	exonic	nonsynonymous SNV	Chr1	2553380	G	T	
125/126	AT1G08140	exonic	nonsynonymous SNV	Chr1	2553393	G	A	
125/126	AT1G08140	exonic	nonsynonymous SNV	Chr1	2554146	C	G	
125/126	AT1G08140	exonic	nonsynonymous SNV	Chr1	2554217	C	A	
125/126	AT1G08140	exonic	nonsynonymous SNV	Chr1	2554240	T	C	
125/126	AT1G08150	exonic	nonsynonymous SNV	Chr1	2556911	A	T	ATCHX5, CATION/H+ EXCHANGER 5, CHX5
125/126	AT1G08150	exonic	nonsynonymous SNV	Chr1	2557414	C	G	
125/126	AT1G08150	exonic	nonsynonymous SNV	Chr1	2558213	G	T	
125/126	AT1G08150	exonic	nonsynonymous SNV	Chr1	2558221	A	C	
125/126	AT1G08150	exonic	nonsynonymous SNV	Chr1	2558270	C	T	
125/126	AT1G08150	exonic	nonsynonymous SNV	Chr1	2558300	T	G	
125/126	AT1G08150	exonic	nonsynonymous SNV	Chr1	2558827	T	A	
125/126	AT1G08150	exonic	nonsynonymous SNV	Chr1	2559028	A	T	
125/126	AT1G08150	exonic	nonsynonymous SNV	Chr1	2559048	C	G	
125/126	AT1G08150	exonic	nonsynonymous SNV	Chr1	2559065	G	T	

125/126	AT1G08170	exonic	nonsynonymous SNV	Chr1	2563240	C	T	Histone superfamily protein
125/126	AT1G08170	exonic	nonsynonymous SNV	Chr1	2563458	A	G	
125/126	AT1G08170	exonic	nonsynonymous SNV	Chr1	2563473	G	C	
125/126	AT1G08170	exonic	nonsynonymous SNV	Chr1	2563515	A	G	
125/126	AT1G08170	exonic	nonsynonymous SNV	Chr1	2563569	A	G	
125/126	AT1G08170	exonic	nonsynonymous SNV	Chr1	2563606	T	C	
125/126	AT1G08190	exonic	nonsynonymous SNV	Chr1	2571385	G	C	
125/126	AT1G08210	exonic	nonsynonymous SNV	Chr1	2577153	A	G	Eukaryotic aspartyl protease family protein
125/126	AT1G08210	exonic	nonsynonymous SNV	Chr1	2577192	T	A	
125/126	AT1G08210	exonic	nonsynonymous SNV	Chr1	2579650	A	C	
125/126	AT1G08220	exonic	nonsynonymous SNV	Chr1	2581872	C	T	ATPase complex subunit
125/126	AT1G08220	exonic	nonsynonymous SNV	Chr1	2582456	C	T	
125/126	AT1G08220	exonic	nonsynonymous SNV	Chr1	2582551	G	A	
125/126	AT1G08230	exonic	nonsynonymous SNV	Chr1	2584326	T	A	Codes for a H+-driven, high affinity gamma-aminobutyric acid (GABA) transporter
125/126	AT1G08230	exonic	nonsynonymous SNV	Chr1	2584662	G	T	
125/126	AT1G08230	exonic	nonsynonymous SNV	Chr1	2584663	G	T	
125/126	AT1G08230	exonic	nonsynonymous SNV	Chr1	2584702	G	A	
125/126	AT1G08230	exonic	nonsynonymous SNV	Chr1	2584705	C	T	
125/126	AT1G08230	exonic	nonsynonymous SNV	Chr1	2586289	T	C	
125/126	AT1G08230	exonic	nonsynonymous SNV	Chr1	2586414	G	T	
125/126	AT1G08250	exonic	nonsynonymous SNV	Chr1	2590091	C	T	
125/126	AT1G08260	exonic	nonsynonymous SNV	Chr1	2604067	A	G	ABA OVERLY SENSITIVE 4, ABO4, EARLY IN SHORT DAYS 7, EMB142, EMB2284, EMB529,
125/126	AT1G08260	exonic	nonsynonymous SNV	Chr1	2605196	C	G	
125/126	AT1G08260	exonic	nonsynonymous SNV	Chr1	2606695	G	C	
125/126	AT1G08270	exonic	nonsynonymous SNV	Chr1	2607078	T	A	
125/126	AT1G08280	exonic	nonsynonymous SNV	Chr1	2608462	G	A	GALT29A, GLYCOSYLTRANSFERASE 29A
125/126	AT1G08280	exonic	nonsynonymous SNV	Chr1	2608534	T	A	
125/126	AT1G08280	exonic	nonsynonymous SNV	Chr1	2608969	A	G	
125/126	AT1G08280	exonic	nonsynonymous SNV	Chr1	2609119	A	G	
125/126	AT1G08280	exonic	nonsynonymous SNV	Chr1	2609290	A	G	
125/126	AT1G08280	exonic	nonsynonymous SNV	Chr1	2609540	G	A	
125/126	AT1G08290	exonic	nonsynonymous SNV	Chr1	2611044	A	G	
125/126	AT1G08290	exonic	nonsynonymous SNV	Chr1	2612922	G	T	

125/126	AT1G08300	exonic	frameshift insertion	Chr1	2616190	C	CT	NO VEIN-LIKE, NVL
125/126	AT1G08300	exonic	frameshift insertion	Chr1	2618749	G	GA	
125/126	AT1G08300	exonic	nonsynonymous SNV	Chr1	2615541	C	A	
125/126	AT1G08300	exonic	nonsynonymous SNV	Chr1	2615681	A	C	
125/126	AT1G08300	exonic	nonsynonymous SNV	Chr1	2616091	T	C	
125/126	AT1G08300	exonic	nonsynonymous SNV	Chr1	2616190	C	T	
125/126	AT1G08300	exonic	nonsynonymous SNV	Chr1	2617758	A	C	
125/126	AT1G08300	exonic	nonsynonymous SNV	Chr1	2617809	T	A	
125/126	AT1G08300	exonic	nonsynonymous SNV	Chr1	2617815	A	G	
125/126	AT1G08300	exonic	nonsynonymous SNV	Chr1	2617873	C	A	
125/126	AT1G08300	exonic	nonsynonymous SNV	Chr1	2617914	A	G	
125/126	AT1G08300	exonic	nonsynonymous SNV	Chr1	2617980	C	T	
125/126	AT1G08300	exonic	nonsynonymous SNV	Chr1	2618297	C	T	
125/126	AT1G08300	exonic	nonsynonymous SNV	Chr1	2618641	T	A	
125/126	AT1G08310	exonic	nonsynonymous SNV	Chr1	2619464	C	A	
125/126	AT1G08320	exonic	nonsynonymous SNV	Chr1	2625734	C	T	
125/126	AT1G08340	exonic	frameshift insertion	Chr1	2632664	C	CA	Rho GTPase activating protein with PAK-box/P21-Rho-binding domain-containing protei
125/126	AT1G08340	exonic	nonsynonymous SNV	Chr1	2632486	T	A	
125/126	AT1G08340	exonic	nonsynonymous SNV	Chr1	2632613	G	T	
125/126	AT1G08350	exonic	nonsynonymous SNV	Chr1	2633817	G	A	
125/126	AT1G08350	exonic	nonsynonymous SNV	Chr1	2635515	C	T	
125/126	AT1G08370	exonic	frameshift insertion	Chr1	2639859	C	CCTCACCAA	
125/126	AT1G08370	exonic	nonsynonymous SNV	Chr1	2639845	A	C	
125/126	AT1G08370	exonic	nonsynonymous SNV	Chr1	2640076	C	G	
125/126	AT1G08390	exonic	nonsynonymous SNV	Chr1	2642583	A	C	
125/126	AT1G08400	exonic	nonsynonymous SNV	Chr1	2644262	A	T	RINT-1 / TIP-1 family
125/126	AT1G08400	exonic	nonsynonymous SNV	Chr1	2644442	G	A	
125/126	AT1G08400	exonic	nonsynonymous SNV	Chr1	2645038	C	T	
125/126	AT1G08400	exonic	nonsynonymous SNV	Chr1	2645213	C	T	
125/126	AT1G08410	exonic	nonsynonymous SNV	Chr1	2646460	A	T	DIG6, DROUGHT INHIBITED GROWTH OF LATERAL ROOTS 6, LSG1-2, YEAST LSG1 ORTHO
125/126	AT1G08410	exonic	nonsynonymous SNV	Chr1	2648160	G	A	
125/126	AT1G08410	exonic	nonsynonymous SNV	Chr1	2648581	A	C	
125/126	AT1G08410	exonic	nonsynonymous SNV	Chr1	2648588	A	C	

125/126	AT1G08420	exonic	nonsynonymous SNV	Chr1	2650127	C	T	
125/126	AT1G08420	exonic	nonsynonymous SNV	Chr1	2653619	A	T	
125/126	AT1G08430	exonic	nonsynonymous SNV	Chr1	2658847	T	G	
125/126	AT1G08430	exonic	nonsynonymous SNV	Chr1	2660670	G	A	
125/126	AT1G08440	exonic	nonsynonymous SNV	Chr1	2664419	C	T	aluminum activated malate transporter family protein
125/126	AT1G08440	exonic	nonsynonymous SNV	Chr1	2664489	C	G	
125/126	AT1G08440	exonic	nonsynonymous SNV	Chr1	2664744	A	T	
125/126	AT1G08440	exonic	nonsynonymous SNV	Chr1	2664756	T	G	
125/126	AT1G08440	exonic	nonsynonymous SNV	Chr1	2664772	C	T	
125/126	AT1G08440	exonic	nonsynonymous SNV	Chr1	2664818	C	A	
125/126	AT1G08440	exonic	nonsynonymous SNV	Chr1	2664873	A	G	
125/126	AT1G08440	exonic	nonsynonymous SNV	Chr1	2665107	G	C	
125/126	AT1G08440	exonic	nonsynonymous SNV	Chr1	2665295	T	G	
125/126	AT1G08460	exonic	nonsynonymous SNV	Chr1	2672698	A	G	ATHDA8, HDA08, HDA8, HISTONE DEACETYLASE 8
125/126	AT1G08460	exonic	nonsynonymous SNV	Chr1	2672729	T	G	
125/126	AT1G08460	exonic	nonsynonymous SNV	Chr1	2673048	G	T	
125/126	AT1G08460	exonic	nonsynonymous SNV	Chr1	2674228	T	C	
125/126	AT1G08460	exonic	nonsynonymous SNV	Chr1	2674356	C	T	
125/126	AT1G08460	exonic	nonsynonymous SNV	Chr1	2674392	G	C	
125/126	AT1G08460	exonic	nonsynonymous SNV	Chr1	2674399	A	T	
125/126	AT1G08460	exonic	nonsynonymous SNV	Chr1	2674429	C	G	
125/126	AT1G08460	exonic	nonsynonymous SNV	Chr1	2674437	A	T	
125/126	AT1G08470	exonic	nonsynonymous SNV	Chr1	2683834	C	A	
125/126	AT1G08470	exonic	nonsynonymous SNV	Chr1	2683974	C	T	
125/126	AT1G08480	exonic	nonsynonymous SNV	Chr1	2684443	G	C	
125/126	AT1G08490	exonic	nonsynonymous SNV	Chr1	2686021	C	A	
125/126	AT1G08490	exonic	nonsynonymous SNV	Chr1	2687526	C	A	
125/126	AT1G08500	exonic	nonsynonymous SNV	Chr1	2689634	C	G	
125/126	AT1G08520	exonic	nonsynonymous SNV	Chr1	2696747	T	A	ALB-1V, ALB1, ALBINA 1, CHLD, PDE166, PIGMENT DEFECTIVE EMBRYO 166, V157
125/126	AT1G08520	exonic	nonsynonymous SNV	Chr1	2697959	A	T	
125/126	AT1G08520	exonic	nonsynonymous SNV	Chr1	2699081	A	C	
125/126	AT1G08530	exonic	frameshift insertion	Chr1	2701364	A	ACT	chitinase-like protein
125/126	AT1G08530	exonic	frameshift insertion	Chr1	2702948	A	AT	

125/126	AT1G08530	exonic	nonsynonymous SNV	Chr1	2701294	T	C	
125/126	AT1G08530	exonic	nonsynonymous SNV	Chr1	2701297	C	T	
125/126	AT1G08530	exonic	nonsynonymous SNV	Chr1	2701309	G	C	
125/126	AT1G08530	exonic	nonsynonymous SNV	Chr1	2701319	C	G	
125/126	AT1G08530	exonic	nonsynonymous SNV	Chr1	2701340	C	A	
125/126	AT1G08530	exonic	nonsynonymous SNV	Chr1	2701366	G	C	
125/126	AT1G08530	exonic	nonsynonymous SNV	Chr1	2701366	G	T	
125/126	AT1G08530	exonic	nonsynonymous SNV	Chr1	2701369	T	A	
125/126	AT1G08530	exonic	nonsynonymous SNV	Chr1	2701370	C	A	
125/126	AT1G08530	exonic	nonsynonymous SNV	Chr1	2701372	C	A	
125/126	AT1G08530	exonic	nonsynonymous SNV	Chr1	2701373	A	T	
125/126	AT1G08530	exonic	nonsynonymous SNV	Chr1	2701376	C	T	
125/126	AT1G08530	exonic	nonsynonymous SNV	Chr1	2701423	A	G	
125/126	AT1G08530	exonic	nonsynonymous SNV	Chr1	2701522	T	G	
125/126	AT1G08530	exonic	nonsynonymous SNV	Chr1	2702918	A	C	
125/126	AT1G08540	exonic	nonsynonymous SNV	Chr1	2703512	A	C	ABC1, ATSIG1, ATSIG2, RNA POLYMERASE SIGMA SUBUNIT 1, RNAPOLYMERASE SIGMA [illegible]
125/126	AT1G08540	exonic	nonsynonymous SNV	Chr1	2704774	T	C	
125/126	AT1G08540	exonic	nonsynonymous SNV	Chr1	2704795	A	T	
125/126	AT1G08540	exonic	nonsynonymous SNV	Chr1	2704796	T	C	
125/126	AT1G08540	exonic	nonsynonymous SNV	Chr1	2704832	A	T	
125/126	AT1G08550	exonic	nonsynonymous SNV	Chr1	2707640	C	T	ARABIDOPSIS VIOLAXANTHIN DE-EPOXIDASE 1, AVDE1, NON-PHOTOCHEMICAL QUENCH[illegible]
125/126	AT1G08550	exonic	nonsynonymous SNV	Chr1	2707773	C	G	
125/126	AT1G08550	exonic	nonsynonymous SNV	Chr1	2709007	A	G	
