# Supplementary material for: The complex genetic architecture of shoot growth natural variation in Arabidopsis thaliana
Source: PLoS Genet. 2019 Apr 22;15(4):e1007954. doi: 10.1371/journal.pgen.1007954 (PMC6476473; doi:10.1371/journal.pgen.1007954)
Supplement: S6 Table — List of Cvi/Col differentially expressed genes in an allele-specific fashion ([log2]>0.8 in at least one condition; from Cubillos et al., 2014) across the whole region. (PDF) [file pgen.1007954.s013.pdf]

Supplementary Table S6: microStairs; differentially expressed genes controlled in cis

List of Cvi/Col differentially expressed genes in an allele-specific fashion ([log2]>0.8 in at least one condition; from Cubillos *et al.*, 2014) across the whole region.

	ASE Control (WW)				ASE Stress (WD)					
Gene ID	Col reads	Cvi reads	Col/Cvi(log2)	q-value	Col reads	Cvi reads	Col/Cvi(log2)	q_value	**intervals microStairs**	**Gene generic name**
AT1G01650	81	108	-0,402639894	0,07560446	108	194	-0,845655158	3,65713E-06	101/102 &102/103	ARABIDOPSIS THALIANA SIGNAL PEPTIDE PEPTIDASE-LIKE 4, ATSPPL4
AT1G02330	349	138	1,337279936	6,97655E-21	332	134	1,307526862	2,08595E-19	103/104 &104/105	COP1 SUPPRESSOR 2, CSU2
**AT1G02820**	187	90	1,056044642	1,7085E-08	209	120	0,80043073	6,11044E-06	105/106 & 106/107	ATLEA3, LATE EMBRYOGENESIS ABUNDANT 3, LEA3
AT1G04270	1232	589	1,064582504	1,12091E-50	1095	562	0,960855303	2,26474E-38	110/111 & 111/112	CYTOSOLIC RIBOSOMAL PROTEIN S15, RPS15
AT1G04590	151	95	0,665060862	0,001028409	238	120	0,984119372	2,61273E-09	111/112 & 112/113	PPR containing-like protein;(source:Araport11)
AT1G04820	946	158	2,579042003	5,8722E-135	1261	267	2,240487717	1,7323E-152	112/113 & 113/114	TOR2, TORTIFOLIA 2, TUA4, TUBULIN ALPHA-4 CHAIN
AT1G05190	757	1326	-0,808945186	8,83925E-35	1285	1744	-0,440815502	7,13456E-16	114/115 & 115/116	EMB2394, EMBRYO DEFECTIVE 2394, RPL6
AT1G05200	387	256	0,600150477	1,02508E-06	274	149	0,880791484	7,1714E-09	114/115 & 115/116	ATGLR3.4, GLR3.4, GLUR3, GLUTAMATE RECEPTOR 3.4
AT1G05830	322	185	0,801618342	6,04279E-09	242	147	0,720931695	5,31431E-06	116/117 & 117/118	ATX2, SDG30, SET DOMAIN PROTEIN 30, TRITHORAX-LIKE PROTEIN 2
**AT1G05940**	314	169	0,896291292	2,26578E-10	287	184	0,645465342	9,71077E-06	117/118 & 118/119	CAT9, CATIONIC AMINO ACID TRANSPORTER 9
**AT1G06010**	316	122	1,369212951	1,11664E-19	116	55	1,076416631	1,41487E-05	117/118 & 118/119	basic leucine zipper/W2 domain protein;(source:Araport11)
AT1G06550	128	238	-0,893177584	5,53467E-08	136	215	-0,657481446	8,27649E-05	118/119 & 119/120	ATP-dependent caseinolytic (Clp) protease/crotonase family protein;(source:Araport11)
**AT1G06640**	849	75	3,500514393	5,4528E-165	1317	119	3,465373996	9,1562E-254	119/120 & 120/121	similar to a 2-oxoglutarate-dependent dioxygenase
AT1G06645	317	185	0,77935644	1,92031E-08	298	114	1,384912101	4,99852E-19	119/120 & 120/121	2-oxoglutarate (2OG) and Fe(II)-dependent oxygenase superfamily protein
AT1G07110	439	267	0,718500344	7,06647E-10	392	221	0,831349133	3,3311E-11	120/121 & 121/122	"FRUCTOSE-2,6-BISPHOSPHATASE", ATF2KP, F2KP, FKFBP
**AT1G07140**	586	205	1,516955755	5,29432E-42	392	188	1,063211171	1,59872E-16	120/121 & 121/122	SIRANBP
AT1G07470	430	165	1,383568313	4,19265E-27	432	173	1,320547496	2,32775E-25	122/123 & 123/124	Transcription factor IIA, alpha/beta subunit;(source:Araport11)
AT1G07670	481	261	0,88332755	4,14228E-15	443	234	0,917972643	6,87879E-15	122/123 & 123/124	ATECA4, ECA4, ENDOMEMBRANE-TYPE CA-ATPASE 4
**AT1G07790**	114	308	-1,438891813	1,055E-20	73	131	-0,842002787	0,000195055	123/124 & 124/125	HTB1
AT1G08000	419	211	0,986977656	6,69742E-16	335	265	0,337338638	0,009191087	123/124 & 124/125	GATA TRANSCRIPTION FACTOR 10, GATA10
**AT1G08650**	670	401	0,738737387	1,41887E-15	1019	383	1,412385267	2,80763E-65	126/127 & 127/128	ATPPCK1, PHOSPHOENOLPYRUVATE CARBOXYLASE KINASE 1, PPCK1
**AT1G09200**	743	241	1,626965603	1,2041E-58	1115	306	1,866440723	2,2682E-106	128/129 & 129/130	H3.1, HISTONE 3.1
AT1G09590	4894	2357	1,054069628	4,3167E-197	4069	1539	1,402271696	5,0292E-257	128/129 & 129/130	Translation protein SH3-like family protein;(source:Araport11)

Extracted from Cubillos et al., The Plant Cell, 2014
Genes that were also identified as local eQTL (Cubillos et al., BMC Genomics, 2012) are in bold
